# Polytope Novikov homology

**DOI:** 10.1007/s11784-021-00899-5

**Published:** 2021-09-24

**Authors:** Alessio Pellegrini

**Affiliations:** Department of Mathematics, ETH Zürich, Zurich, Switzerland

**Keywords:** 57R58, 53D40, 37D15, 55Nxx

## Abstract

Let *M* be a closed manifold and ${\mathcal {A}} \subseteq H^1_{\mathrm {dR}}(M)$ a polytope. For each $a \in {\mathcal {A}}$, we define a Novikov chain complex with a multiple finiteness condition encoded by the polytope ${\mathcal {A}}$. The resulting polytope Novikov homology generalizes the ordinary Novikov homology. We prove that any two cohomology classes in a prescribed polytope give rise to chain homotopy equivalent polytope Novikov complexes over a Novikov ring associated with said polytope. As applications, we present a novel approach to the (twisted) Novikov Morse Homology Theorem and prove a new polytope Novikov Principle. The latter generalizes the ordinary Novikov Principle and a recent result of Pajitnov in the abelian case.

## Introduction

Given a closed manifold *M* and a cohomology class $a \in H^1_{\mathrm {dR}}(M)$, one can define the so-called *Novikov homology* $\mathrm {HN}_\bullet (a)$, introduced by Novikov [11, 12]. Roughly speaking, $\mathrm {HN}_\bullet (a)$ is defined by picking a Morse representative $\alpha \in a$ and a cover on which $\alpha$ pulls back to an exact form $d{\tilde{f}}$, and then mimicking the definition of Morse homology using ${\tilde{f}}$ as the underlying Morse function. The groups $\mathrm {HN}_\bullet (a)$ enjoy three distinctive features

- **(Novikov module)** The Novikov homology $\mathrm {HN}_\bullet (a)$ is a finitely generated module over the so-called *Novikov ring* $\mathrm {Nov}(a)$.
- **(Cohomology invariance)** The Novikov homology $\mathrm {HN}_\bullet (a)$ does not depend on the choice of Morse representative $\alpha$ of the prescribed cohomology class *a*.
- **(Ray invariance)** Morse forms on the same positive half-ray induce identical Novikov homologies: $\mathrm {HN}_\bullet (r \cdot a) \cong \mathrm {HN}_\bullet (a)$ for all $r>0$.

The (twisted) Novikov Morse Homology Theorem says that $\mathrm {HN}_\bullet (a)$ is isomorphic to the twisted singular homology $H_\bullet \left( M,\underline{\mathrm {Nov}}(a)\right)$.^1^ Using the Novikov Morse Homology Theorem, one can thus investigate the relation between $\mathrm {HN}_\bullet (a)$ and $\mathrm {HN}_\bullet (b)$, when $a \ne b$, by studying $H_\bullet \left( M,\underline{\mathrm {Nov}}(a)\right)$ and $H_\bullet \left( M,\underline{\mathrm {Nov}}(b)\right)$ and their respective twisted coefficient systems $\underline{\mathrm {Nov}}(a)$ and $\underline{\mathrm {Nov}}(b)$ instead. The latter is a “purely" algebraic task.

In this article, we refine the construction of Novikov homology $\mathrm {HN}_\bullet (a)$ and define what we call *polytope Novikov homology* $\mathrm {HN}_\bullet (a,{\mathcal {A}})$ by including multiple finiteness conditions imposed by a polytope ${\mathcal {A}}=\langle a_0,\dots , a_k \rangle \subseteq H^1_{\mathrm {dR}}(M)$ containing *a*. These polytope Novikov homology groups $\mathrm {HN}_\bullet (a,{\mathcal {A}})$ retain the three features of $\mathrm {HN}(a)$ mentioned above, modulo replacing $\mathrm {Nov}(a)$ by a “smaller" Novikov ring $\mathrm {Nov}({\mathcal {A}})$. The Main Theorem in Sect. 2 gives a *dynamical* relation between $\mathrm {HN}_\bullet (a,{\mathcal {A}})$ and $\mathrm {HN}_\bullet (b,{\mathcal {A}})$, i.e., by staying in the realm of Novikov homology and not resorting to the algebraic counterpart of twisted singular homology.

### Theorem

(Main Theorem^2^) For every subpolytope ${\mathcal {B}} \subseteq {\mathcal {A}}$ and two cohomology classes $a,b \in {\mathcal {A}}$, there exists a commutative diagram 
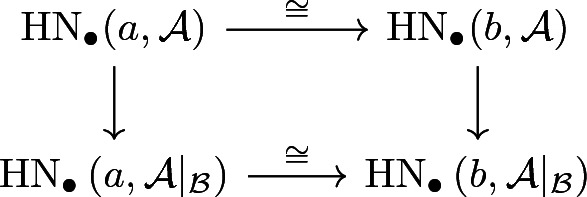
 induced by continuation on the chain level.

The statement of the Main Theorem might be known to some experts in the field, but lacks a proof in the literature. Similar variants of the Main Theorem have been proved in different settings; most noteworthy are [5, 7, 13, 25]. For example, in [13], Ono considers Novikov–Floer homology on a closed symplectic manifold^3^ and proves the following.

### Theorem

(Ono [13]) If two symplectic isotopies have fluxes that are close to each other, then their respective Novikov–Floer homologies are isomorphic.

The Novikov–Floer homologies mentioned in Ono’s Theorem are defined over a common Novikov ring that takes into account several finiteness conditions simultaneously—this modification is analogous to our implementation of polytopes. Within this analogy, the upper isomorphism in the Main Theorem corresponds to the isomorphism in Ono’s Theorem, but with less assumptions: the nearby assumption of the fluxes in Ono’s result would translate to a smallness assumption on ${\mathcal {A}}$, which is not needed here. Let us mention that the formulation and setup of the Main Theorem comes closest to a recent result due to Groman and Merry [5, Theorem 5.1].

At the end of the paper, we present two applications of the Main Theorem. In the first application, we recover the aforementioned Novikov Morse Homology Theorem:^4^ The proof, modulo details, goes as follows: taking ${\mathcal {A}}=\langle 0,a \rangle$, setting ${\mathcal {B}}=\langle a \rangle$, invoking the lower isomorphism in the Main Theorem, and unwinding the definitions reveals

$$\begin{aligned} \mathrm {HN}_\bullet (a) \cong \mathrm {HM}_\bullet (f,\underline{ \mathrm {Nov}}(a)) , \end{aligned}$$

where the right-hand side is Morse homology with local coefficients $\underline{\mathrm {Nov}}(a)$. The latter is known to be isomorphic to singular homology with twisted coefficients, for a quick proof, see [1, Theorem 4.1], and thus, we recover the Novikov Morse Homology Theorem. This line of reasoning is analogous to the proof of [5, Theorem 5.3] and seems to be a novel approach to the Novikov Morse Homology Theorem: the proof draws a direct connection between Novikov and *twisted* Morse homology instead of using the Novikov Principle and/or equivariant Morse homology; see [4, 8, 19] for proofs of the Novikov Morse Homology Theorem using the latter.

The second application is concerned with a general *polytope Novikov Principle*:^5^

### Theorem

(Polytope Novikov Principle) Let ${\mathcal {B}}\subseteq {\mathcal {A}}$ be a subpolytope. Then, for every $a \in {\mathcal {A}}$, there exists a Morse representative $\alpha \in a$, such that

$$\begin{aligned} \mathrm {CN}_\bullet \left( \alpha ,{\mathcal {A}} |_{{\mathcal {B}}} \right) \simeq C_\bullet \left( {\widetilde{M}}_{\mathcal {A}}\right) \otimes _{{\mathbb {Z}}[\Gamma _{\mathcal {A}}]}\mathrm {Nov}({\mathcal {A}}|_{\mathcal {B}}), \end{aligned}$$

as Novikov modules.

The proof idea is similar to the sketch above—one relates the polytope Novikov complex to a twisted Morse complex by including the 0-vertex in the polytope ${\mathcal {A}}$ and using the Main Theorem. We call this the *0-vertex trick* (cf. Lemma 3.1). To get from the twisted Morse complex to the equivariant singular chain complex, we use a Morse–Eilenberg type result (cf. Lemma 3.2) and a chain homotopy equivalence $\mathrm {CM}({\tilde{h}}) \simeq C_\bullet \left( {\widetilde{M}}_{{\mathcal {A}}} \right)$ over the group ring of deck transformations ${\mathbb {Z}}[\Gamma _{{\mathcal {A}}}]$.

Immediate consequences of the polytope Novikov Principle include the ordinary Novikov Principle (cf. Corollary 3.8) and a recent “conical" Novikov Principle [17, Theorem 5.1]^6^ in the abelian case (cf. Corollary 3.10).

### Remark

Symplectic homology is a version of Floer homology well suited to certain non-compact symplectic manifolds. In [18], we combine ideas of Ono’s Theorem, the magnetic case [5], and of the present paper to construct a polytope Novikov symplectic homology, which is related to Ritter’s twisted symplectic homology [20]. The analogue of the Main Theorem remains true. Applications include Novikov number-type bounds on the number of fixed points of symplectomorphisms with prescribed flux on the boundary, and the study of symplectic isotopies of such maps.

## Novikov homology and polytopes

### Definition and properties of ordinary Novikov homology

In this subsection, we quickly recall the (ordinary) definition of the Novikov chain complex and its homology, together with some well known properties. The main purpose is to fix the notation for the remainder of the section. For a thorough treatment of Novikov homology, we recommend [4, 19, 22] and the recently published [1]. For more details on the construction of the Novikov ring, see for instance [6, Chapter 4].

Fix once and for all a closed smooth oriented and connected finite-dimensional manifold *M*. For any Morse–Smale pair $(\alpha ,g)$ one can define the *Novikov chain complex*

$$\begin{aligned} (\mathrm {CN}_\bullet (\alpha ,g), \partial _\bullet ), \end{aligned}$$

whose homology is called *Novikov homology* of $(\alpha ,g)$It is a standard fact that two Morse–Smale pairs with cohomologous Morse forms induce isomorphic Novikov homologies, and thus, we shall write $\mathrm {HN}_\bullet (a)$ with $a=[\alpha ]$ to denote the Novikov homology of pairs $(\alpha ,g)$.

**Notation**. Sometimes, we will also omit the *g* in the notation of the chain complex. Moreover, Latin lowercase letters, e.g., *a*, *b*, will typically denote cohomology classes, while the respective lowercase Greek letters are representatives in the corresponding cohomology classes, e.g., $\alpha \in a, \, \beta \in b$.

Let us quickly recall the relevant definitions. Each cohomology class *a* determines a period homomorphism $\Phi _a :\pi _1(M) \rightarrow {\mathbb {R}}$ defined by integrating any representative $\alpha \in a$ over loops $\gamma$ in *M*.^7^ Denote by $\ker (a)$ the kernel of the period homomorphism $\Phi _a$ and let $\pi :{\widetilde{M}}_a \rightarrow M$ be the associated abelian cover, i.e., a regular covering with . Then, $\alpha$ pulls back to an exact form on ${\widetilde{M}}_a$, i.e., $\pi ^*\alpha =d {\tilde{f}}_\alpha$ for some ${\tilde{f}}_\alpha \in C^{\infty }({\widetilde{M}}_a)$. Define

$$\begin{aligned} V_i(\alpha ):= \bigoplus _{{\tilde{x}} \in \mathrm {Crit}_i({\tilde{f}}_\alpha )} {\mathbb {Z}}\langle {\tilde{x}} \rangle , \quad i \in \mathbb {N}_0, \end{aligned}$$

where $\mathrm {Crit}_i({\tilde{f}}_\alpha )$ denotes the critical points of ${\tilde{f}}_\alpha$ with Morse index *i*. The *i*th Novikov chain group $\mathrm {CN}_i(\alpha )$ can then be defined as the *downward completion* of $V_i(\alpha )$ with respect to ${\tilde{f}}_\alpha$, which shall be denoted by

1 $$\begin{aligned} {\widehat{V}}_i(\alpha )_{{\tilde{f}}_\alpha } \text { or more concisely } {\widehat{V}}_i(\alpha )_\alpha . \end{aligned}$$

Explicitly, elements $\xi \in \mathrm {CN}_i(\alpha )$ are infinite sums with a *finiteness condition* determined by ${\tilde{f}}_\alpha$:

$$\begin{aligned} \xi =\sum _{{\tilde{x}} \in \mathrm {Crit}_i({\tilde{f}}_{\alpha })} \xi _{{\tilde{x}}} \, {\tilde{x}} \in \mathrm {CN}_i(\alpha ) \; \iff \; \forall c \in {\mathbb {R}}:\; \lbrace {\tilde{x}} \, \big | \, \xi _{{\tilde{x}}} \ne 0 \in {\mathbb {Z}}, \; {\tilde{f}}_{\alpha }({\tilde{x}})>c \rbrace \text { is finite}. \end{aligned}$$

The boundary operator is defined by counting Novikov–Morse trajectories of ${\tilde{f}}_{\alpha }$

$$\begin{aligned} \partial :\mathrm {CN}_i(\alpha ) \rightarrow \mathrm {CN}_{i-1}(\alpha ), \quad \partial \xi :=\sum _{{\tilde{x}}, \, {\tilde{y}}} \, \xi _{{\tilde{x}}} \cdot \#_{\mathrm {alg}} \, \underline{{\mathcal {M}}}({\tilde{x}},{\tilde{y}};{\tilde{f}}_{\alpha }) \, {\tilde{y}}, \end{aligned}$$

where

$$\begin{aligned} {\mathcal {M}}({\tilde{x}},{\tilde{y}};{\tilde{f}}_{\alpha })=\lbrace {\tilde{\gamma }} \in C^{\infty }({\mathbb {R}},{\widetilde{M}}) \, \big | \, \dot{{\tilde{\gamma }}}+\nabla ^{{\tilde{g}}} {\tilde{f}}_{\alpha }({\tilde{\gamma }})=0, \, {\tilde{\gamma }}(-\infty )={\tilde{x}}, \, {\tilde{\gamma }}(+\infty )={\tilde{y}} \rbrace \end{aligned}$$

is the usual moduli space with ${\tilde{g}}=\pi ^*g$ the pullback metric. Denote . Similarly, we denote by ${\mathcal {M}}(x,y;\alpha )$ and $\underline{{\mathcal {M}}}(x,y;\alpha )$ the moduli spaces downstairs. The $\#_{\mathrm {alg}}$ indicates the algebraic count, i.e., counting the Novikov–Morse trajectories with signs determined by a choice of orientation of the underlying unstable manifolds.

The *Novikov ring* $\Lambda _\alpha$ associated with $\alpha \in a$ is defined as the *upward completion* of the group ring ${\mathbb {Z}}[\Gamma _a]$ with respect to the period homomorphism $\Phi _a$, and therefore

$$\begin{aligned} \lambda =\sum _{A \in \Gamma _a} \lambda _A \, A \in \Lambda _\alpha \; \iff \; \forall c \in {\mathbb {R}}:\; \lbrace A \, \big | \, \lambda _A \ne 0 \in {\mathbb {Z}}, \; \Phi _a(A) < c \rbrace \text { is finite}. \end{aligned}$$

The Novikov ring $\Lambda _\alpha$ does not depend on the choice of representative $\alpha \in a$, and thus, we shall write $\Lambda _a$. Moreover, $\Lambda _a$ acts on $\mathrm {CN}_\bullet (\alpha )$ in the obvious way. By fixing a preferred lift ${\tilde{x}}_j$ in each fiber of the finitely many zeros $x_j \in Z(\alpha ):=\left\{ x \in M \, \big | \, \alpha (x)=0 \right\}$, one can view $\mathrm {CN}_\bullet (\alpha )$ as a finitely generated $\Lambda _a$-module

2 $$\begin{aligned} \mathrm {CN}_i(\alpha ) \cong \bigoplus _{x_j \in Z(\alpha )} \Lambda _a \langle {\tilde{x}}_j \rangle \, \text { as Novikov ring modules}. \end{aligned}$$

Another standard fact asserts that the boundary operator $\partial$ is $\Lambda _a$-linear, and consequently, the Novikov homology $\mathrm {HN}_\bullet (a)$ carries a $\Lambda _a$-module structure. The latter is implicitly using the fact that isomorphism of Novikov homologies for cohomologous Morse forms, which is suppressed in the notation $\mathrm {HN}_\bullet (a)$, is also $\Lambda _a$-linear.

#### Remark 2.1

If *M* is *not* orientable, one can still define a Novikov homology by replacing ${\mathbb {Z}}$ with ${\mathbb {Z}}_2$ in all the definitions above.

### Novikov homology with polytopes

We are now ready to refine the Novikov chain complex using polytopes—this notion is key for the proofs of all incoming theorems.

#### Definition 2.2

Given $a_0,\dots ,a_k \in H^1_{\mathrm {dR}}(M)$, denote by

$$\begin{aligned} {\mathcal {A}}=\langle a_0, \dots , a_k \rangle \subset H^1_{\mathrm {dR}}(M) \end{aligned}$$

the **polytope** spanned by the **vertices** $\{a_l\}_{l=0,\dots ,k}$, i.e., the set of all convex combinations

$$\begin{aligned} a=\sum _{l=0}^k c_l \cdot a_l \text { with } c_l \in [0,1] \text { and } \sum _{l=0}^k c_l=1. \end{aligned}$$

To any polytope ${\mathcal {A}}$, we associate a regular coverand we shall abbreviate

$$\begin{aligned} \Gamma _{\mathcal {A}}:=\mathrm {Deck}({\widetilde{M}}_{{\mathcal {A}}}). \end{aligned}$$

#### Example 2.3

For the polytope ${\mathcal {A}}=\langle a \rangle$, the covering ${\widetilde{M}}_{\mathcal {A}}$ agrees with the abelian cover ${\widetilde{M}}_a$ associated with $a \in H^1_{\mathrm {dR}}(M)$. The same is true for any polytope ${\mathcal {A}}$, whose other vertices $a_l$ satisfy $\ker (a) \subseteq \ker (a_l)$.

The defining condition of ${\widetilde{M}}_{{\mathcal {A}}}$ ensures that each vertex $a_l$ pulls back to the trivial cohomology class, and so does every $a \in {\mathcal {A}}$; see Lemma 2.7. We write ${\tilde{f}}_{\alpha } \in C^{\infty }({\widetilde{M}}_{{\mathcal {A}}})$ to denote some primitive of $\pi ^*\alpha$ for $\alpha$ a representative of $a \in {\mathcal {A}}$. Now, we fix a smooth section

$$\begin{aligned} \theta :{\mathcal {A}} \longrightarrow \Omega ^1(M), \quad a \mapsto \theta _a \end{aligned}$$

of the projection of closed one-forms to their cohomology class. In other words, $\theta _a$ is a representative of *a*. This enables us to talk about a “preferred" representative of each cohomology class in the polytope.

For every polytope $a \in {\mathcal {A}}$, we define

$$\begin{aligned} V_i(\theta _a,{\mathcal {A}}):= \bigoplus _{{\tilde{x}} \in \mathrm {Crit}_i\left( {\tilde{f}}_{\theta _a}\right) } {\mathbb {Z}}\langle {\tilde{x}} \rangle . \end{aligned}$$

The subtle but crucial difference to $V_i(\theta _a)$ is that ${\widetilde{M}}_{{\mathcal {A}}}$ does not necessarily coincide with the abelian cover ${\widetilde{M}}_a$.

#### Definition 2.4

Let ${\mathcal {A}}$ be a polytope with section $\theta :{\mathcal {A}} \rightarrow \Omega ^1(M)$. Then, the **(polytope) Novikov chain complex groups**

$$\begin{aligned} \mathrm {CN}_i(\theta _a,{\mathcal {A}}), \quad i \in {\mathbb {N}}_0, \end{aligned}$$

are defined as the intersections of the downward completions of $V_i(\theta _a,{\mathcal {A}})$ with respect to any ${\tilde{f}}_{\beta } :{\widetilde{M}}_{{\mathcal {A}}} \rightarrow {\mathbb {R}}$ for $b \in {\mathcal {A}}$. In other words, with the notation of (1)

$$\begin{aligned} \mathrm {CN}_i(\theta _a,{\mathcal {A}}):=\bigcap _{b \in {\mathcal {A}}} {\widehat{V}}_i(\theta _a,{\mathcal {A}})_{\beta }. \end{aligned}$$

#### Remark 2.5

Let $\beta \in b$ be any representative. The choice of primitive ${\tilde{f}}_{\beta }$ of $\pi ^*\beta$ is unique up to adding constants and hence does not affect the finiteness condition. Additionally, two primitives ${\tilde{f}}_{\beta }$ and ${\tilde{f}}_{\beta '}$ induce the same finiteness condition for $\beta , \, \beta ' \in b$. Indeed, ${\tilde{f}}_{\beta '}-{\tilde{f}}_{\beta }=h \circ \pi$ for some smooth $h :M \rightarrow {\mathbb {R}}$ with $dh=\beta '-\beta$. Since *M* is compact, we get

$$\begin{aligned} {\tilde{f}}_{\beta }({\tilde{x}})>c \implies {\tilde{f}}_{\beta '}({\tilde{x}})> \min _{z} h(z) +c \text { and } {\tilde{f}}_{\beta '}({\tilde{x}})>d \implies {\tilde{f}}_{\beta }({\tilde{x}}) > d-\max _{z} h(z)l \end{aligned}$$

two finiteness conditions are equivalent. This justifies Definition 2.4.

Unpacking Definition 2.4, we see

3 $$\begin{aligned} \xi= & {} \sum _{{\tilde{x}} \in \mathrm {Crit}_i\left( {\tilde{f}}_{\theta _a}\right) } \xi _{{\tilde{x}}} \, {\tilde{x}} \in \mathrm {CN}_i(\theta _a, {\mathcal {A}}) \iff \forall b \in {\mathcal {A}}, \forall c \in {\mathbb {R}}:\; \nonumber \\&\#\lbrace {\tilde{x}} \, \big | \, \xi _{{\tilde{x}}} \ne 0, \; {\tilde{f}}_\beta ({\tilde{x}})>c \rbrace < +\infty , \end{aligned}$$

where it does not matter which primitives ${\tilde{f}}_\beta$ we use, cf. Remark 2.5. The right-hand side describes a finiteness condition that has to hold for all $b \in {\mathcal {A}}$, and hence, we will refer to it as the *multi finiteness condition*.

**Notation**. In view of Remark 2.5, we shall write

$$\begin{aligned} \mathrm {CN}_i(\theta _a,{\mathcal {A}})=\bigcap _{b \in {\mathcal {A}}} {\widehat{V}}_i(\theta _a,{\mathcal {A}})_b \end{aligned}$$

from now on.

In a similar fashion, we can define yet another completion of $V_i(\theta _a,{\mathcal {A}})$ by taking the completion with respect to less one-forms.

#### Definition 2.6

Let ${\mathcal {B}} \subseteq {\mathcal {A}}$ be a subpolytope, i.e., the convex hull of a subset of the vertices of ${\mathcal {A}}$. Then, we define

$$\begin{aligned} \mathrm {CN}_i\left( \theta _a,{\mathcal {A}} |_{{\mathcal {B}}}\right) :=\bigcap _{b \in {\mathcal {B}}} {\widehat{V}}_i(\theta _a,{\mathcal {A}})_b \end{aligned}$$

the **restricted** (polytope) Novikov chain complex groups of ${\mathcal {B}}\subseteq {\mathcal {A}}$.

By definition, we get the inclusion

$$\begin{aligned} \mathrm {CN}_\bullet (\theta _a,{\mathcal {A}}) \subseteq \mathrm {CN}_\bullet \left( \theta _a, {\mathcal {A}} |_{{\mathcal {B}}} \right) , \text { for all subpolytopes } {\mathcal {B}} \subseteq {\mathcal {A}}. \end{aligned}$$

The next lemma asserts that $\mathrm {CN}_\bullet (\theta _a,{\mathcal {A}})$ is uniquely determined by the vertices of ${\mathcal {A}}$. In other words, one only needs to check the multi-finiteness condition for the finitely many vertices $a_l$. This is a straightforward adaptation of [25, Lemma 7.3].

#### Lemma 2.7

Let $\theta :{\mathcal {A}} \rightarrow \Omega ^1(M)$ be as above. Then

$$\begin{aligned} \mathrm {CN}_i(\theta _a, {\mathcal {A}})=\bigcap _{b \in {\mathcal {A}}} {\widehat{V}}_i(\theta _a,{\mathcal {A}})_b=\bigcap _{l=0}^k {\widehat{V}}_i(\theta _a,{\mathcal {A}})_{a_l}=\bigcap _{l=0}^k\mathrm {CN}_i \left( \theta _a, {\mathcal {A}} |_{a_l}\right) , \quad \forall a \in {\mathcal {A}}. \end{aligned}$$

More generally, for every subpolytope ${\mathcal {B}} \subseteq {\mathcal {A}}$ spanned by $b_j=a_{l_j}$

$$\begin{aligned} \mathrm {CN}_i(\theta _a, {\mathcal {A}} |_{{\mathcal {B}}})=\bigcap _{j}\mathrm {CN}_i \left( \theta _a, {\mathcal {A}} |_{b_j}\right) , \quad \forall a \in {\mathcal {A}}. \end{aligned}$$

One can play a similar game with the Novikov rings:

#### Definition 2.8

Define the **(polytope) Novikov ring**

$$\begin{aligned} \Lambda _{\mathcal {A}}=\bigcap _{b \in {\mathcal {A}}} {\widehat{{\mathbb {Z}}}}[\Gamma _{{\mathcal {A}}}]^b, \end{aligned}$$

where ${\widehat{{\mathbb {Z}}}}[\Gamma _{{\mathcal {A}}}]^b$ denotes the upward completion of the group ring ${\mathbb {Z}}[\Gamma _{{\mathcal {A}}}]$ with respect to the period homomorphism $\Phi _b :\Gamma _{{\mathcal {A}}} \rightarrow {\mathbb {R}}$. Analogously, for every subpolytope ${\mathcal {B}} \subseteq {\mathcal {A}}$, we define the **restricted** polytope Novikov ring

$$\begin{aligned} \Lambda _{{\mathcal {A}} |_{{\mathcal {B}}}}=\bigcap _{b \in {\mathcal {B}}} {\widehat{{\mathbb {Z}}}}[\Gamma _{{\mathcal {A}}}]^b. \end{aligned}$$

As before, we get

$$\begin{aligned} \Lambda _{{\mathcal {A}}} \subseteq \Lambda _{{\mathcal {A}} |_{{\mathcal {B}}}}, \text { for all subpolytopes } {\mathcal {B}} \subseteq {\mathcal {A}}. \end{aligned}$$

The obvious analogue to Lemma 2.7 holds for Novikov rings as well. These rings enable us to view the polytope Novikov chain complexes as finite Novikov modules just as in the ordinary setting (2).

Next, we try to equip the groups $\mathrm {CN}_\bullet (\theta _a,{\mathcal {A}})$ with a boundary operators that turns them into a genuine chain complex. The obvious candidate would be

4 $$\begin{aligned} \partial _{\theta _a} :\mathrm {CN}_\bullet (\theta _a,{\mathcal {A}}) \rightarrow \mathrm {CN}_{\bullet -1}(\theta _a,{\mathcal {A}}), \quad \partial _{\theta _a} \xi := \sum _{{\tilde{x}}, {\tilde{y}}} \xi _{{\tilde{x}}} \cdot \#_{\mathrm {alg}} \, \underline{{\mathcal {M}}}\left( {\tilde{x}},{\tilde{y}};{\tilde{f}}_{\theta _a}\right) \, {\tilde{y}}.\nonumber \\ \end{aligned}$$

Note that the moduli space above actually also depends on a choice of metric *g*, and so does the boundary operator $\partial _{\theta _a}$. When we want to keep track of the metric, we will write $\mathrm {CN}_\bullet (\theta _a,g,{\mathcal {A}})$. For restrictions ${\mathcal {A}} |_{{\mathcal {B}}}$, we define the boundary operator analogously.

Formally, the definition of $\partial _{\theta _a}$ looks identical to the definition of $\partial$ on $\mathrm {CN}_\bullet (\alpha )$, and morally it is. However, there are two major differences. First, the cover ${\widetilde{M}}_{\mathcal {A}}$ might differ from the abelian cover ${\widetilde{M}}_a$ of *a*. Second, it is not clear whether $\partial =\partial _{\theta _a}$ preserves the multi finiteness condition, i.e., whether $\partial \xi$ lies in $\mathrm {CN}_\bullet (\theta _a,{\mathcal {A}})$. Luckily, we will achieve this by replacing the original section $\theta$ with a perturbed section $\vartheta :{\mathcal {A}} \rightarrow \Omega ^1(M)$ (cf. Theorem 2.14). Whenever the chain complex is defined, we make the following definition.

#### Definition 2.9

Let $\vartheta :{\mathcal {A}} \rightarrow \Omega ^1(M)$ be a section, such that $\left( \mathrm {CN}_\bullet (\vartheta _a,g_{\vartheta _a},{\mathcal {A}}),\partial \right)$ defines a chain complex. Then, we call the induced homology **(polytope) Novikov homology** and denote it by

$$\begin{aligned} \mathrm {HN}_\bullet (\vartheta _a,g_{\vartheta _a},{\mathcal {A}}) \text { or more abusively }\mathrm {HN}_\bullet (\vartheta _a,{\mathcal {A}}). \end{aligned}$$

Analogously, we define

$$\begin{aligned} \mathrm {HN}_\bullet \left( \vartheta _a,g_{\vartheta _a},{\mathcal {A}} |_{{\mathcal {B}}} \right) =\mathrm {HN}_\bullet \left( \vartheta _a,{\mathcal {A}} |_{{\mathcal {B}}}\right) . \end{aligned}$$

#### Remark 2.10

Analogously to ordinary Novikov homology, one can show that the Novikov homologies $\mathrm {HN}_\bullet (\vartheta _a,{\mathcal {A}})$ and $\mathrm {HN}_\bullet \left( \vartheta _a,{\mathcal {A}} |_{{\mathcal {B}}} \right)$ are both finitely generated modules over the Novikov rings $\Lambda _{\mathcal {A}}$ and $\Lambda _{{\mathcal {A}} |_{{\mathcal {B}}}}$, respectively, thus generalizing the Novikov-module property. This follows from the fact that the boundary operator (4) is $\Lambda _{{\mathcal {A}}}$-linear (and similarly for the restricted case).

### Technical results for Sect. 2.4

In this subsection, we state and prove all the technical auxiliary results needed for the proof of Theorem 2.14, which roughly speaking asserts the well-definedness of the polytope chain complexes and their respective homologies after modifying the section $\theta :{\mathcal {A}} \rightarrow \Omega ^1(M)$ to a new section $\vartheta :{\mathcal {A}} \rightarrow \Omega ^1(M)$.

**Notation.** For any (closed) one-form $\rho$, we will denote by $\nabla ^g \rho$ the dual vector field to $\rho$ with respect to the metric *g*. Note that with this notation, we have $\nabla ^g H=\nabla ^g dH$ for any smooth function $H :M \rightarrow {\mathbb {R}}$.

#### Proposition 2.11

Let $(\rho ,g)$ be a Morse–Smale pair. Then, for every $\delta >0$, there exists a constant $C_\rho =C_\rho (\delta ,g)>0$, such that

$$\begin{aligned} \Vert \nabla ^g \rho (z) \Vert< C_\rho \implies \exists x \in Z(\rho ) \text { with } d(x,z)<\delta , \end{aligned}$$

where both $\Vert \cdot \Vert$ and $d( \, \cdot \, , \, \cdot \, )$ are induced by *g*.

#### Proof

Suppose the assertion does not hold. Then, there exists a $\delta >0$, a positive sequence $C_k \rightarrow 0$, and $(z_k) \subset M$, such that

$$\begin{aligned} \Vert \nabla ^g \rho (z_k) \Vert < C_k \quad \text {and} \quad z_k \in M \setminus \bigcup _{x \in Z(\rho )}B_\delta (x). \end{aligned}$$

By compactness of *M*, we can pass to a subsequence $(z_k)$ converging to some $z \in M$. The above however implies $\Vert \nabla ^g \rho (z) \Vert =0$, which is equivalent to $z \in Z(\rho )$. At the same time, *z* lies in $M \setminus \bigcup _{x \in Z(\rho )}B_\delta (x)$, which is a contradiction. This concludes the proof. $\square$

**Notation.** Such a constant $C_\rho >0$ is often referred to as a *Palais–Smale constant* (short: *PS-constant*). The main case of interest is the exact one, i.e., $\rho =dH$, for which we will abbreviate $C_{dH}=C_H$. Sometimes, we will also abbreviate $C_{\rho }=C$.

The next lemma builds the main technical tool of Sect. 2.4. The idea is to perturb one-forms $\alpha$ close to a given *reference Morse–Smale pair* $(\rho ,g)$, so that the pertubations, say $\alpha '$, maintain their cohomology classes of $\alpha$, become Morse, have the same zeros as $\rho$, and are still relatively close to $\rho$. This is reminiscent of Zhang’s arguments [25, Section 3].

#### Lemma 2.12

Let $(\rho ,g)$ be a Morse–Smale pair, $\delta >0$ so small that the balls $B_{2\delta }(x)$, with $x \in Z(\rho )$, are geodesically convex^8^ and lie in pairwise disjoint charts of *M*, and $C=C_\rho (\delta ,g)>0$ as in Proposition 2.11.

Let $$\alpha \in \Omega ^1(M)$$ with

$$\begin{aligned} \Vert \alpha - \rho \Vert < \frac{C}{8} \quad \text {and}\quad a=[\alpha ], \end{aligned}$$

where $\Vert \cdot \Vert$ is the norm induced by *g*. Then, there exists a Morse–Smale pair $(\alpha ',g')$, with $\alpha ' \in a$, satisfying

- $$\Vert \alpha ' - \rho \Vert \le 5 \cdot \Vert \alpha -\rho \Vert $$,
for all $x \in Z(\rho )$ and
- $$Z(\alpha ')=Z(\rho )$$.

Moreover, $\Vert \nabla ^{g'} \alpha '(z) \Vert ' < \frac{C}{8}$ implies $z \in B_\delta (x)$^9^ for some zero $x \in Z(\alpha ')$, where $\Vert \cdot \Vert '$ is the norm induced by $g'$.

#### Proof

Since $\rho$ is a Morse form, there are only finitely many zeros $x \in Z(\rho )$. Around each such *x*, we will perturb $\alpha$ without changing its cohomology class: enumerate the finitely many zeros of $\rho$ by $\{x_i\}_{i=1,\dots ,k}$ and pick positive bump functions $h_i :M \rightarrow {\mathbb {R}}_{\ge 0}$ with

$$\begin{aligned} {\left\{ \begin{array}{ll} h_i \equiv 0, &{}\text { on } M\setminus B_{2\delta }(x_i) \\ h_i \equiv 1, &{}\text { on } B_\delta (x_i) \\ \Vert \nabla ^g h_i \Vert \le \frac{2}{\delta }. \end{array}\right. } \end{aligned}$$

Since every $B_{2\delta }(x_i)$ is simply connected, there exist unique smooth functions $f_i :B_{2\delta }(x_i) \rightarrow {\mathbb {R}}$ satisfying5We set

6 $$\begin{aligned} \alpha '=\alpha - \sum _{i=1}^k d(h_i \cdot f_i). \end{aligned}$$

By construction, we have $\alpha ' \in a$, $\alpha '=\rho$ on $B_\delta (x)$ for $x \in Z(\rho )$, and that $\alpha '$ agrees with $\alpha$ outside of $\bigcup _{i=0}^k B_{2\delta }(x_i)$. Consequently, $\alpha '-\rho$ and $\alpha -\rho$ agree outside of $\bigcup _{i=0}^k B_{2\delta }(x_i)$. This means that for the inequality in the first bullet point, it suffices to argue why the bound holds inside each ball $B_{2 \delta }(x_i)$. Inserting the definitions grants

$$\begin{aligned} \Vert \alpha ' -\rho \Vert _{B_{2\delta }(x_i)}&= \Vert \alpha - \rho - h_i \cdot df_i - f_i \cdot dh_i \Vert _{B_{2\delta }(x_i)} \\&\le (1-h_i) \Vert \alpha -\rho \Vert _{B_{2\delta }(x_i)}+\Vert f_i \Vert _{B_{2\delta }(x_i)} \cdot \Vert \nabla ^g h_i \Vert _{B_{2\delta }(x_i)} \\&\le \Vert \alpha -\rho \Vert _{B_{2\delta }(x_i)} + \frac{2}{\delta } \cdot \Vert f_i \Vert _{B_{2\delta }(x_i)}. \end{aligned}$$

Recall that $f_i$ was chosen, such that $f_i(x_i)=0$. Due to the geodesic convexity of the balls $B_{2\delta }(x_i)$, we can apply the mean value inequality

$$\begin{aligned} \vert f_i(y) \vert= & {} \vert f_i(x_i)-f_i(y) \vert \le \Vert \nabla ^g f_i \Vert _{B_{2\delta }(x_i)} \cdot d(x_i,y) \le \Vert \alpha -\rho \Vert _{B_{2\delta }(x_i)} \cdot 2\delta , \\&\quad \forall y \in B_{2\delta }(x_i). \end{aligned}$$

All in all, this implies

$$\begin{aligned} \Vert \alpha '-\rho \Vert _{B_{2\delta }(x_i)} \le \Vert \alpha - \rho \Vert _{B_{2\delta }(x_i)}+ \frac{4\delta }{\delta } \Vert \alpha -\rho \Vert _{B_{2\delta }(x_i)} =5 \cdot \Vert \alpha -\rho \Vert _{B_{2\delta }(x_i)}. \end{aligned}$$

This proves the first inequality in the first bullet point. From this, we will deduce that $Z(\rho )=Z(\alpha ')$: the inclusion $Z(\rho )\subseteq Z(\alpha ')$ is clear as $\rho$ agrees with $\alpha '$ around $Z(\rho )$. The reverse inclusion is obtained by observing that for $y \in Z(\alpha ')$, we have

$$\begin{aligned} \Vert \nabla ^g \rho (y) \Vert = \Vert \nabla ^g \rho (y)-\underbrace{\nabla ^g\alpha '(y)}_{=0} \Vert \le \Vert \rho - \alpha ' \Vert \le 5 \cdot \Vert \alpha - \rho \Vert < C, \end{aligned}$$

by assumption on $\rho$ and the inequality above. Proposition 2.11 then implies that *z* has to be a zero of $\rho$, as well. This proves $Z(\rho )=Z(\alpha ')$, in particular that $\alpha '$ is a Morse form.

To get a Riemannian metric $g'$ that turns $(\alpha ',g')$ into a Morse–Smale pair, it suffices to perturb *g* on an open set that intersects all the Novikov–Morse trajectories of $(\alpha ',g)$; see [19, Page 38–40] for more details. Since $Z(\rho )=Z(\alpha ')$, we can take a perturbation $g'$ that agrees with *g* on $M \setminus \bigcup _{i=1}^k B_\delta (x_i)$ and is close to *g* in the $C^{\infty }$-topology.

The last assertion of the statement follows from the observation that, for $\alpha '$ fixed, the map $g' \mapsto \Vert \nabla ^{g'} \alpha ' \Vert '$ is continuous, and thus, for $g'$ close to *g*, we get

$$\begin{aligned} \Vert \nabla ^g \alpha '(z) \Vert \le \underbrace{ \vert \Vert \nabla ^g \alpha '(z) \Vert - \Vert \nabla ^{g'} \alpha '(z) \Vert ' \vert }_{ < \varepsilon } + \Vert \nabla ^{g'} \alpha '(z) \Vert '. \end{aligned}$$

Assuming $\Vert \nabla ^{g'}\alpha '(z) \Vert ' < \frac{C}{8}$, we thus end up with

$$\begin{aligned} \Vert \nabla ^g \rho (z) \Vert&\le \Vert \nabla ^g \rho (z) - \nabla ^g \alpha '(z) \Vert + \Vert \nabla ^g \alpha '(z) \Vert \\&\le \Vert \rho -\alpha ' \Vert + \varepsilon + \frac{C}{8}&\text { using the above inequality}, \\&\le 5 \cdot \Vert \rho - \alpha \Vert + \varepsilon + \frac{C}{8}&\text { by the first bullet point}, \\&< \frac{5 \cdot C}{8}+ \varepsilon +\frac{C}{8}&\text { by assumption.} \end{aligned}$$

Taking $\varepsilon \le \frac{C}{4}$ and invoking Proposition 2.11 then conclude the proof. $\square$

Lemma 2.12 can be applied to a whole section $\theta :{\mathcal {A}} \rightarrow \Omega ^1(M)$ *nearby* a reference Morse–Smale pair $(\rho ,g)$ and give rise to a perturbed section $\vartheta :{\mathcal {A}} \rightarrow \Omega ^1(M)$ that is still relatively close to $\rho$, so that each $\vartheta _a$ agrees with $\rho$ near the zeros $x \in Z(\rho )$.

#### Proposition 2.13

Let $(\rho ,g)$ and $C=C_{\rho }>0$ as in Lemma 2.12, denote $N=\bigcup _{i} B_{2\delta }(x_i)$ with $x_i \in Z(\rho )$, and let $\theta :{\mathcal {A}} \rightarrow \Omega ^1(M)$ be a section satisfying

7 $$\begin{aligned} \Vert \theta _a-\rho \Vert < \frac{C}{8}. \end{aligned}$$

Then, there exists a section

$$\begin{aligned} \vartheta =\vartheta (\theta ,\rho ,g) :{\mathcal {A}} \longrightarrow \Omega ^1(M) \end{aligned}$$

and a positive constant $D=D(N,g)>0$ with the following significance:^10^

- $$Z(\vartheta _a)=Z(\rho )$$ for all $$a \in {\mathcal {A}}$$,
for all $$x_i \in Z(\rho ), \, a \in {\mathcal {A}}$$.

Moreover, for every $\vartheta _a$, there exists a Riemannian metric $g_{\vartheta _a}$ close to *g* with , such that

- $(\vartheta _a,g_{\vartheta _a})$ is Morse–Smale,
- $$\Vert \vartheta _b-\rho \Vert _{\vartheta _a} \le D \cdot \Vert \vartheta _b- \rho \Vert \le 5 \cdot D \cdot \Vert \theta _b-\rho \Vert $$ for all $$a,b \in {\mathcal {A}},$$

where $\Vert \cdot \Vert _{\vartheta _a}$ is the operator norm induced by $g_{\vartheta _a}$.

#### Proof

Since the whole section $\theta :{\mathcal {A}} \rightarrow \Omega ^1(M)$ is $\frac{C}{8}$-close to $(\rho ,g)$, we can take $(\rho ,g)$ as a reference pair and apply Lemma 2.12 to every $\theta _a$ and denote $\vartheta _a$ the corresponding perturbation. Recall from the proof of Lemma 2.12 that $\vartheta _a$ is obtained by an exact perturbation of $\theta _a$ around the zeros of $\rho$—a closer inspection reveals that this exact perturbation varies smoothly along $\theta _a$, in particular that $\vartheta$ defines a smooth section. The first two bullet points follow immediately from Lemma 2.12.

We choose $g_{\vartheta _a}=g_{(\theta _a)'}$ just as $g'$ in Lemma 2.12, i.e., by means of a small perturbation of *g* inside *N*. The argument in [19] shows that sufficiently small perturbations give rise to Riemannian metrics that are *uniformly* equivalent to the original *g*; in other words, we may choose $g_{\vartheta _a}$, such that $(\vartheta _a,g_{\vartheta _a})$ is Morse–Smale and

$$\begin{aligned} \frac{1}{D^2} g_{\vartheta _a}(v,v) \le g(v,v) \le D^2g_{\vartheta _a}(v,v), \quad \forall v \in TM, \, \forall a \in {\mathcal {A}}, \end{aligned}$$

with $D>0$ a constant that only depends on *N* and *g*. Using this inequality and invoking, the first bullet point of Lemma 2.12 conclude the proof. $\square$

### Section perturbations

We can finally state and prove Theorem 2.14 by applying the previous results in the special case of *exact* reference pairs:

#### Theorem 2.14

Let $\theta :{\mathcal {A}} \rightarrow \Omega ^1(M)$ be a section and (*H*, *g*) a reference Morse–Smale pair on *M*. Then, there exists a perturbed section

$$\begin{aligned} \vartheta =\vartheta (\theta ,H,g) :{\mathcal {A}} \longrightarrow \Omega ^1(M) \end{aligned}$$

and a choice of Riemannian metrics $g_{\vartheta _a}$ with the following significance:

- **(Morse–Smale property)** Each pair $(\vartheta _a,g_{\vartheta _a})$ is Morse–Smale, for all $a \in {\mathcal {A}}$;
- **(Chain complex)** The chain complex $\left( \mathrm {CN}_\bullet \left( \vartheta _a,g_{\vartheta _a},{\mathcal {A}} \right) ,\partial _{\vartheta _a} \right)$ is well defined for every pair $(\vartheta _a,g_{\vartheta _a})$ as above;
- **(Ray invariance)** The chain complexes are equal upon scaling, i.e., $\mathrm {CN}_\bullet \left( \vartheta _a,g_{\vartheta _a},{\mathcal {A}} \right) =\mathrm {CN}_\bullet \left( r \cdot \vartheta _a, g_{\vartheta _a}, r \cdot {\mathcal {A}} \right)$ for all $r>0$, $a \in {\mathcal {A}}$.^11^

The rough idea is to *“shift-and-scale"*: we shift and scale the polytope ${\mathcal {A}}$, so that it is sufficiently close to a given exact one-form *dH* in the operator norm $\Vert \cdot \Vert$ coming from *g*. Then, one can perturb the scaled section by means of Proposition 2.13 and scale back. This will be the desired section $\vartheta$ on ${\mathcal {A}}$. By construction, we will then see that the three bullet points are satisfied. The choices involved (i.e., choice of section $\theta$, reference pair (*H*, *g*), and perturbation coming from Theorem 2.14) will prove harmless—they result in chain homotopy equivalent complexes. This is proven in the next subsection (cf. Theorem 2.19).

At the cost of imposing a smallness condition on the underlying section, we get the same results for perturbations associated with non-exact reference pairs (cf. Corollary 2.17) and the same independence of auxiliary data holds (cf. Theorem 2.22).

#### Proof of Theorem 2.14

As a first candidate for $\vartheta$, we pick

$$\begin{aligned} \vartheta :{\mathcal {A}} \longrightarrow \Omega ^1(M), \quad \vartheta _a:= \theta _a + dH. \end{aligned}$$

This is still a section, but does not satisfy the bullet points above. Since $\theta$ is smooth, there exists

$$\begin{aligned} \varepsilon =\varepsilon (\theta ,H,g,\delta )>0, \end{aligned}$$

such that

$$\begin{aligned} \varepsilon \cdot \theta _a+dH \text { is } \frac{C_H}{D \cdot 1000}\text {-close to } dH, \end{aligned}$$

with respect to $\Vert \cdot \Vert$ induced by *g*, $C_H=C_H(\delta ,g)>0$ and $D=D(N,g)>0$ chosen as in Proposition 2.13. Now, we can apply Proposition 2.13 to the section $\varepsilon \cdot a \mapsto \varepsilon \cdot \theta _a+dH$ and obtain a new section^12^

$$\begin{aligned} \vartheta ^{\varepsilon } :\varepsilon \cdot {\mathcal {A}} \longrightarrow \Omega ^1(M).^{12}. \end{aligned}$$

Finally, we scale back and redefine

$$\begin{aligned} \vartheta =\vartheta (\theta ,H,g,\varepsilon ) :{\mathcal {A}} \longrightarrow \Omega ^1(M), \quad a \mapsto \frac{1}{\varepsilon } \cdot \vartheta ^{\varepsilon }(\varepsilon \cdot a). \end{aligned}$$

Thus, we have

$$\begin{aligned} \varepsilon \cdot \vartheta _a=\vartheta ^{\varepsilon }(\varepsilon \cdot a), \quad \forall a \in {\mathcal {A}}. \end{aligned}$$

For each $\vartheta ^{\varepsilon }(\varepsilon \cdot a)$, we choose a Riemannian metric denoted by $g_{a}$ as in Proposition 2.13. Thus, $(\vartheta ^{\varepsilon }(\varepsilon \cdot a),g_{a})$ is Morse–Smale, and so is $(\vartheta _a,g_{a})$, since scaling does not affect the Morse–Smale property. This proves the first bullet point.

#### Claim

$\mathrm {CN}_\bullet \left( \vartheta ^{\varepsilon }(\varepsilon \cdot a), \varepsilon \cdot {\mathcal {A}} \right) =\mathrm {CN}_\bullet (\varepsilon \cdot \vartheta _a,\varepsilon \cdot {\mathcal {A}})$ is a well-defined chain complex for any $a \in {\mathcal {A}}$.

Indeed, assume for contradiction that there exists a Novikov chain $\xi =\sum _{{\tilde{x}}} \xi _{{\tilde{x}}} \, {\tilde{x}} \in \mathrm {CN}_\bullet \left( \vartheta ^{\varepsilon }(\varepsilon \cdot a), \varepsilon \cdot {\mathcal {A}} \right)$, such that

$$\begin{aligned} \partial \xi \notin \mathrm {CN}_{\bullet -1}\left( \vartheta ^{\varepsilon }(\varepsilon \cdot a), \varepsilon \cdot {\mathcal {A}} \right) . \end{aligned}$$

This means that there are some $\varepsilon \cdot b \in \varepsilon \cdot {\mathcal {A}}$, $c \in {\mathbb {R}}$ and sequences ${\tilde{x}}_n$ with $\xi _{{\tilde{x}}_n} \ne 0$, ${\tilde{y}}_n$ pairwise distinct, ${\tilde{\gamma }}_n \in \mathcal {{\underline{M}}}\left( {\tilde{x}}_n,{\tilde{y}}_n;{\tilde{f}}_{\varepsilon \cdot \vartheta _a}\right)$, and

$$\begin{aligned} {\tilde{f}}_{\vartheta ^{\varepsilon }(\varepsilon \cdot b)}({\tilde{y}}_n)={\tilde{f}}_{\varepsilon \cdot \vartheta _b}({\tilde{y}}_n) \ge c; \end{aligned}$$

see Remark 2.5. Denote by $\gamma _n=\pi \circ {\tilde{\gamma }}_n$ the Novikov–Morse trajectories downstairs. The energy expression can then be massaged as follows:

$$\begin{aligned} 0 \le E({\tilde{\gamma }}_n)=E(\gamma _n)&=-\int _{\gamma _n} \vartheta ^{\varepsilon }(\varepsilon \cdot a) \\&=-\int _{\gamma _n} \vartheta ^{\varepsilon }(\varepsilon \cdot b) + \int _{\gamma _n} \vartheta ^{\varepsilon }(\varepsilon \cdot b)-\vartheta ^{\varepsilon }(\varepsilon \cdot a) \\&={\tilde{f}}_{\vartheta ^{\varepsilon }(\varepsilon \cdot b)}({\tilde{x}}_n)-{\tilde{f}}_{\vartheta ^{\varepsilon }(\varepsilon \cdot b)}({\tilde{y}}_n)+\int _{\gamma _n} \vartheta ^{\varepsilon }(\varepsilon \cdot b)-\vartheta ^{\varepsilon }(\varepsilon \cdot a) \\&\le {\tilde{f}}_{\vartheta ^{\varepsilon }(\varepsilon \cdot b)}({\tilde{x}}_n)-c+\int _{\gamma _n} \vartheta ^{\varepsilon }(\varepsilon \cdot b)-\vartheta ^{\varepsilon }(\varepsilon \cdot a). \end{aligned}$$

Showing that the rightmost term is bounded by $m \cdot E(\gamma _n), \, m \in (0,1)$ suffices to obtain a contradiction: admitting such a bound leads to

$$\begin{aligned} 0 \le E(\gamma _n) \le (1-m)^{-1} \cdot \left( {\tilde{f}}_{\vartheta ^{\varepsilon }(\varepsilon \cdot b)}({\tilde{x}}_n) - c \right) . \end{aligned}$$

In particular, $c \le {\tilde{f}}_{\vartheta ^{\varepsilon }(\varepsilon \cdot b)}({\tilde{x}}_n)$ for all *n*. However, $\xi$ belongs to $\mathrm {CN}_\bullet \left( \varepsilon \cdot \vartheta _a, \varepsilon \cdot {\mathcal {A}} \right)$ and $\xi _{{\tilde{x}}_n} \ne 0$, and thus, the multi-finiteness condition implies that there are only finitely many distinct ${\tilde{x}}_n$. Up to passing to a subsequence, we can therefore assume ${\tilde{x}}_n={\tilde{x}}$ and also ${\tilde{y}}_n \in \pi ^{-1}(y)$.^13^ The corresponding Novikov–Morse trajectories

$$\begin{aligned} \gamma _n \in \mathcal {{\underline{M}}}\left( x,y;\vartheta ^{\varepsilon }(\varepsilon \cdot a)\right) \end{aligned}$$

have uniformly bounded energy

$$\begin{aligned} E(\gamma _n) \le (1-m)^{-1} \cdot \left( {\tilde{f}}_{\vartheta ^{\varepsilon }(\varepsilon \cdot b)}({\tilde{x}})-c \right) ; \end{aligned}$$

therefore, $\gamma _n$ has a $C^{\infty }_{\mathrm {loc}}$-convergent subsequence. At the same time $\mathcal {{\underline{M}}}\left( x,y;\vartheta ^{\varepsilon }(\varepsilon \cdot a)\right)$ is a 0-dimensional manifold, which means that the convergent subsequence $\gamma _n$ eventually does not depend on *n*. This contradicts our assumption that the endpoints ${\tilde{y}}_n$ upstairs are pairwise disjoint.

Therefore, we are only left to show the bound

$$\begin{aligned} A(\gamma _n):=\int _{\gamma _n} \vartheta ^{\varepsilon }(\varepsilon \cdot b)-\vartheta ^{\varepsilon }(\varepsilon \cdot a) \le \frac{1}{2}E(\gamma _n) \end{aligned}$$

to conclude the Claim. For this purpose, we define

$$\begin{aligned} {\mathcal {S}}_n:= \left\{ s \in {\mathbb {R}}\, \big | \, \Vert \nabla ^{g_{a}} \left( \vartheta ^{\varepsilon }(\varepsilon \cdot a) \right) (\gamma _n(s)) \Vert _{g_{a}} \ge \frac{C_H}{8} \right\} . \end{aligned}$$

The crucial observation is that both $\vartheta ^{\varepsilon }(\varepsilon \cdot b)$ and $\vartheta ^{\varepsilon }(\varepsilon \cdot a)$ agree with *dH* around $\mathrm {Crit}(H)$, by choice of $\vartheta ^{\varepsilon }$ via Proposition 2.13. In particularLemma 2.12 says that for $s \in {\mathbb {R}}\setminus {\mathcal {S}}_n$, we get

$$\begin{aligned} \gamma _n(s) \in \bigcup _{z \in Z(\vartheta ^{\varepsilon }(\varepsilon \cdot a))}B_\delta (z); \end{aligned}$$

consequently

8 $$\begin{aligned} \int _{{\mathbb {R}}\setminus {\mathcal {S}}_n} \left( \vartheta ^{\varepsilon }(\varepsilon \cdot b)-\vartheta ^{\varepsilon }(\varepsilon \cdot a)\right) \, {\dot{\gamma }}_n(s) \, \mathrm{d}s=0. \end{aligned}$$

The Lebesgue measure $\mu ({\mathcal {S}}_n)$ can be bounded using the energy

$$\begin{aligned} E(\gamma _n)=-\int _{\gamma _n} \vartheta ^{\varepsilon }(\varepsilon \cdot a) =\int _{\mathbb {R}}\Vert \nabla ^{g_{a}} \left( \vartheta ^{\varepsilon }(\varepsilon \cdot a) \right) (\gamma _n(s)) \Vert _{g_{a}}^2 \, \mathrm{d}s \ge \mu ({\mathcal {S}}_n) \cdot \left( \frac{C_H}{8} \right) ^2; \end{aligned}$$

thus

9 $$\begin{aligned} \mu ({\mathcal {S}}_n) \le \left( \frac{8}{C_H} \right) ^2 \cdot E(\gamma _n). \end{aligned}$$

And finally

$$\begin{aligned} \vert A(\gamma _n) \vert&\le \Vert \vartheta ^{\varepsilon }(\varepsilon \cdot b)-\vartheta ^{\varepsilon }(\varepsilon \cdot a) \Vert _{g_{a}} \cdot \int _{{\mathcal {S}}_n} \Vert {\dot{\gamma }}_n(s) \Vert _{g_{a}} \, \mathrm{d}s \qquad \text { by } (8), \\&\le \big (\Vert \vartheta ^{\varepsilon }(\varepsilon \cdot b)- dH \Vert _{g_{a}} \\&\quad + \Vert dH-\vartheta ^{\varepsilon }(\varepsilon \cdot a) \Vert _{g_{a}} \big ) \cdot \mu ({\mathcal {S}}_n)^{\frac{1}{2}} \cdot E(\gamma _n)^{\frac{1}{2}} \\&\le 5 D \cdot \big (\Vert \varepsilon \cdot \vartheta _b- dH \Vert \\&\quad +\Vert dH-\varepsilon \cdot \vartheta _a \Vert \big ) \cdot \frac{8}{C_H} \cdot E(\gamma _n) \quad \text { Proposition } 2.13, \, (9), \\&\le \frac{80 \cdot D \cdot C_H}{D \cdot 1000 \cdot C_H} \cdot E(\gamma _n) \quad \text { by choice of scaling } \varepsilon >0, \\&<\frac{1}{10}E(\gamma _n). \end{aligned}$$

This proves the Claim.

Now, we observe that scaling $\vartheta _a$ by $r>0$ does not affect the zeros and that the moduli spaces associated with $(\vartheta _a,g_{a})$ are in one-to-one correspondence with those of $(r \cdot \vartheta _a,g_{a})$. It is also clear that the multi- finiteness condition imposed by ${\mathcal {A}}$ is equivalent to that of $r \cdot {\mathcal {A}}$. All in all, this means that for any $r>0$, the polytope chain complexes associated with $(r \cdot \vartheta _a,g_{a})$ agree with each other. This proves the ray invariance. Setting $r=\frac{1}{\varepsilon }$ and using the Claim prove the remaining first bullet point. $\square$

#### Remark 2.15

Instead of running the argument for the sections $\vartheta ^{\varepsilon } :\varepsilon \cdot {\mathcal {A}} \rightarrow \Omega ^1(M)$, we could also work with $\vartheta =\frac{1}{\varepsilon } \cdot \vartheta ^{\varepsilon } :{\mathcal {A}} \rightarrow \Omega ^1(M)$ by directly by applying Proposition 2.13 to the section

$$\begin{aligned} a \mapsto \theta _a+ d\left( \varepsilon ^{-1}H\right) \end{aligned}$$

and $(\varepsilon ^{-1}H,g)$. These two approaches are equivalent; the only difference is psychological: we find it more natural to visualize the shrinking of the polytope opposed to the scaling of Morse functions. Note that the analogous bound at the end of the proof of Theorem 2.14 holds upon replacing *H* by $\varepsilon ^{-1}H$. This follows from the nice scaling behavior of the PS constants:

$$\begin{aligned} C_{\varepsilon ^{-1}H}(\delta ,g)=\varepsilon ^{-1}C_H(\delta ,g); \end{aligned}$$

therefore, “$\varepsilon \cdot \theta _a+dH$ is $\frac{C_H}{D \cdot 1000}$-close to *dH*" if and only if “$\theta _a + d(\varepsilon ^{-1}H)$ is $\frac{C_{\varepsilon ^{-1}H}}{D \cdot 1000}=\frac{C_H}{\varepsilon \cdot D \cdot 1000}$-close to $d(\varepsilon ^{-1}H)$".

#### Remark 2.16

The skeptical reader might wonder whether $\partial _{\vartheta _a}^2=0$ really holds. Viewing the chain group $\mathrm {CN}_\bullet (\vartheta _a,{\mathcal {A}})$ as a certain twisted chain group allows for a quick and simple proof—see Remark 2.35.

The key in the proof of Theorem 2.14 was to obtain control over the energy by perturbing the section $\theta :{\mathcal {A}} \rightarrow \Omega ^1(M)$ via Proposition 2.13. The perturbation was chosen, so that there would be no contribution to the energy near the zeros. Similar ideas to control the energy can be found in [2, Subsection 3.6.2], [25].

The question remains why we used an (exact) reference pair (*H*, *g*) instead of a more general Morse–Smale pair $(\rho ,g)$ in Theorem 2.14. The answer is simple: the given argument already breaks down in the very first line—the corresponding $\vartheta$ is not a section anymore, since the $\rho$-shift changes the cohomology class. However, whenever the section $\theta :{\mathcal {A}} \rightarrow \Omega ^1(M)$ is already *sufficiently close* to $(\rho ,g)$ in terms of the corresponding PS-constant $C_\rho >0$, we do not need to shift and scale $\theta$, and can perturb $\theta$ directly:

#### Corollary 2.17

Let $(\rho ,g)$ be a Morse–Smale pair, $C=C_\rho >0$, $N=\bigcup _i B_{2\delta }(x_i)$ with $x_i \in Z(\rho )$, and $D=d(N,\delta )>0$ as in Proposition 2.13. Let $\theta :{\mathcal {A}} \rightarrow \Omega ^1(M)$ be a smooth section, such that

$$\begin{aligned} \Vert \theta _a-\rho \Vert < \frac{C_\rho }{D \cdot 1000 }, \end{aligned}$$

with $\Vert \cdot \Vert$ the operator norm induced by *g*. Then, there exists a perturbed section

10 $$\begin{aligned} \vartheta =\vartheta (\theta ,\rho ,g) :{\mathcal {A}} \longrightarrow \Omega ^1(M), \end{aligned}$$

and $g_{\vartheta _a}$, such that the same conclusions as in Theorem 2.14 hold.

#### Proof

Upon replacing $\varepsilon \cdot \theta _a+dH$ and *dH* with $\theta _a$ and $\rho$, the proof is word for word the same as the one of Theorem 2.14. $\square$

### Independence of the data

The section $\vartheta =\vartheta (\theta ,H,g)$ constructed in Theorem 2.14 does not only depend on $(\theta ,H,g)$, but also comes with a choice of scaling $\varepsilon (\theta ,H,g,\delta )>0$. We shall prove that any valid perturbation $\vartheta ^i=\vartheta ^i(\theta _i,H_i,g_i,\varepsilon _i)$ in the sense of Theorem 2.14 gives rise to chain homotopy equivalent chain complexes. The same is true for perturbations coming from Corollary 2.17, and at the end of the subsection, we will show that both perturbations lead to chain homotopy equivalent Novikov complexes.

#### Remark 2.18

All the chain maps and chain homotopy equivalences constructed from here on are Novikov-module morphisms, i.e., linear over the Novikov ring. We will not explicitly state this every time for better readability.

#### Theorem 2.19

For $i=0,1$, let $\theta _i :{\mathcal {A}} \rightarrow \Omega ^1(M)$ be sections, $(H_i,g_i)$ Morse–Smale pairs and $\delta _i>0, \, \varepsilon _i(\theta _i,H_i,g_i,\delta _i)>0$ as in proof of Theorem 2.14.

Then any two perturbed sections

11 $$\begin{aligned} \vartheta ^i=\vartheta ^i(\theta _i,H_i,g_i,\varepsilon _i) :{\mathcal {A}} \rightarrow \Omega ^1(M), \end{aligned}$$

in the sense of Theorem 2.14, induce chain homotopy equivalent polytope complexes

12 $$\begin{aligned} \mathrm {CN}_\bullet (\vartheta ^0_a,{\mathcal {A}}) \simeq \mathrm {CN}_\bullet (\vartheta ^1_a,{\mathcal {A}}), \quad \forall a \in {\mathcal {A}}. \end{aligned}$$

#### Proof of Theorem 2.19

We may assume $\varepsilon _1 \ge \varepsilon _0$. Denote by

$$\begin{aligned} \vartheta ^i(\sigma _i,H_i,g_i,\varepsilon _i), \quad i=0,1, \end{aligned}$$

the respective sections on ${\mathcal {A}}$ as in the first part of the proof of Theorem 2.14. Let

13 $$\begin{aligned} h :{\mathbb {R}}\rightarrow {\mathbb {R}}_{\ge 0} \end{aligned}$$

be a positive smooth function with $h \equiv 0$ on $(-\infty ,e)$ and $h\equiv 1$ on $(1-e,+\infty )$, for some small $e>0$, and set

$$\begin{aligned} \vartheta ^s&=(1-h(s)) \cdot \vartheta ^0 +h(s) \cdot \vartheta ^1. \end{aligned}$$

Fix $a \in {\mathcal {A}}$ and pick $g_i:=g_{\vartheta ^i_a}$, $i=0,1$ two metrics as in Theorem 2.14. Let $g_s=g_s(a)$ be a homotopy of Riemannian metrics connecting $g_0$ to $g_1$ and assume that $(\vartheta ^s_a,g_s)$ is regular—this is rectified by Remark 2.20. Note that here $g_s$ actually depends on *a*.

To this regular homotopy, we can now associate a chain continuation

14 $$\begin{aligned} \Psi ^{10} :\mathrm {CN}_\bullet (\vartheta ^0_a,{\mathcal {A}}) \longrightarrow \mathrm {CN}_\bullet (\vartheta ^1_a,{\mathcal {A}}), \quad \xi =\sum _{{\tilde{x}}} \xi _{{\tilde{x}}} \, {\tilde{x}} \mapsto \sum _{{\tilde{x}},{\tilde{y}}} \xi _{{\tilde{x}}} \cdot \#_{\mathrm {alg}} \, {\mathcal {M}}\left( {\tilde{x}},{\tilde{y}};{\tilde{f}}_{\vartheta ^s_a}\right) \, {\tilde{y}}.\nonumber \\ \end{aligned}$$

Analogously to the case of the boundary operator in Theorem 2.14, proving that $\Psi ^{10}$ defines a well-defined Novikov chain map essentially boils down to proving that it respects the multi-finiteness condition—the rest follows by standard Novikov–Morse techniques. Thus, proceeding as in Theorem 2.14 reveals that it suffices^14^ to bound

15 $$\begin{aligned} \int _{\gamma _n} \vartheta ^s_b-\vartheta ^s_a \end{aligned}$$

by either a multiple $m \in (0,1)$ of the energy $E(\gamma _n)$, where *b* is some cohomology class in ${\mathcal {A}}$, or a uniform bound^15^ altogether. Set

$$\begin{aligned} {\mathcal {S}}_n^0&:=\ \left\{ s \in (-\infty ,0) \, \big | \,\Vert \nabla ^{g_0} \vartheta ^0_a (\gamma _n(s)) \Vert _{g_0} \ge \frac{C_H}{\varepsilon _0 \cdot 8} \right\} , \\ {\mathcal {S}}_n^1&:=\ \left\{ s \in (1,+\infty ) \, \big | \,\Vert \nabla ^{g_1} \vartheta ^1_a (\gamma _n(s)) \Vert _{g_1} \ge \frac{C_H}{\varepsilon _1 \cdot 8} \right\} . \end{aligned}$$

This time around we need to divide by $\varepsilon _i$ as we are running the continuation directly on the original polytope ${\mathcal {A}}$ instead of the scaled polytope (see Remark 2.15). As in the previous proof of Theorem 2.14, the $s \in {\mathbb {R}}_{\le 0}\setminus {\mathcal {S}}_n^0$ and $s \in {\mathbb {R}}_{\ge 1} \setminus {\mathcal {S}}_n^1$ do not contribute to (15) as $\gamma _n(s)$ will be near the zeros of $H_i$, where $\vartheta ^i_b=\vartheta ^i_a$. On the other hand, using similar arguments, we obtain

$$\begin{aligned} \bigg \vert \int _{{\mathcal {S}}_n^0 \cup {\mathcal {S}}_n^1} \left( \vartheta ^s_b- \vartheta ^s_a\right) \, {\dot{\gamma }}_n(s) \, \mathrm{d}s \bigg \vert&\le 2\cdot \max _{i=0,1} \Vert \vartheta ^i_b-\vartheta ^i_a \Vert _i \cdot \mu ({\mathcal {S}}^i_n)^{\frac{1}{2}} \cdot E(\gamma _n)^{\frac{1}{2}} \\&\le 2\cdot \max _{i=0,1} \left( \Vert \vartheta ^i_b-dH_i \Vert _i+ \Vert dH_i - \vartheta ^i_a \Vert _i \right) \cdot \frac{\varepsilon _i \cdot 8}{C_H} \cdot E(\gamma _n)\\&\le \max _{i=0,1} \frac{ 4 \cdot 5 \cdot D \cdot \varepsilon _i \cdot 8 \cdot C_H }{D \cdot \varepsilon _i \cdot 1000 \cdot C_H} \cdot E(\gamma _n) \\&\le \frac{1}{5} \cdot E(\gamma _n), \end{aligned}$$

where we have used Proposition 2.13 as in Theorem 2.14. We are left to bound (15) for $s \in [0,1]$. For this, we compute via Cauchy–Schwarz

$$\begin{aligned} \bigg \vert \int _{[0,1]} \left( \vartheta ^s_b- \vartheta ^s_a\right) \, {\dot{\gamma }}_n(s) \, \mathrm{d}s \bigg \vert&\le \max _{s \in [0,1]} \Vert \vartheta ^s_b-\vartheta ^s_a \Vert _s \cdot E(\gamma _n)^{\frac{1}{2}}. \end{aligned}$$

By compactness of $[0,1], \, {\mathcal {A}}$ and continuity of $\vartheta :[0,1] \times {\mathcal {A}} \rightarrow \Omega ^1(M)$, we may bound

$$\begin{aligned} \max _{s \in [0,1]} \Vert \vartheta ^s_b-\vartheta ^s_a \Vert _s \le F, \end{aligned}$$

where $F>0$ is a uniform constant in $s \in [0,1]$ and $b \in {\mathcal {A}}$—recall that $g_s$ depends on *a*, but that does not matter. In particular, this proves

$$\begin{aligned} \bigg \vert \int _{\gamma _n} \vartheta ^s_b-\vartheta ^s_a \, \bigg \vert \le F \cdot E(\gamma _n)^{\frac{1}{2}}+\frac{1}{5} \cdot E(\gamma _n), \quad \forall n \in {\mathbb {N}}, b \in {\mathcal {A}}. \end{aligned}$$

A case distinction now does the job: for any $n \in {\mathbb {N}}$, we either have $\frac{1}{5}E(\gamma _n) \ge F \cdot E(\gamma _n)^{\frac{1}{2}}$ or $\frac{1}{5}E(\gamma _n) < F \cdot E(\gamma _n)^{\frac{1}{2}}$. In the first case, we can bound the norm of (15) by $\frac{2}{5} \cdot E(\gamma _n)$, whereas in the second case, we get $\frac{1}{5} \cdot E(\gamma _n)^{\frac{1}{2}}<F$, and thus, we may bound the norm of (15) by $10F^2$. This proves

$$\begin{aligned} \bigg \vert \int _{\gamma _n} \vartheta ^s_b-\vartheta ^s_a \bigg \vert \le \max \left\{ 10F^2, \frac{2}{5}E(\gamma _n) \right\} . \end{aligned}$$

As explained before, this suffices to conclude that $\Psi ^{10}$ defines a well-defined Novikov chain map, which defines the desired chain homotopy equivalence (see proof of Theorem 2.14 for more details). This concludes the proof. $\square$

#### Remark 2.20

The (linear) homotopy $(\vartheta ^s_a,g_s)$ chosen in the proof of Theorem 2.19 might be non-regular. One can replace $(\vartheta ^s_a,g_s)$ with an arbitrarily close regular homotopy $((\vartheta ^s_a)',g_s')$ connecting the same data. The only bit where this affects the previous argument in Theorem 2.19 is when trying to bound $\max _{s \in [0,1]} \Vert \vartheta ^s_b-(\vartheta ^s_a)' \Vert _s'$. Using that $\vartheta ^s_a$ is smooth in *s* and close to $(\vartheta ^s_a)'$, we still get the desired uniform bound *b*.

As a consequence of Theorems 2.14 and 2.19, we obtain the analogue results for restrictions to subpolytopes ${\mathcal {B}}\subseteq {\mathcal {A}}$:

#### Corollary 2.21

Let $\theta :{\mathcal {A}} \rightarrow \Omega ^1(M)$ be a section. Then, for every perturbed section $\vartheta :{\mathcal {A}} \rightarrow \Omega ^1(M)$ coming from Theorem 2.14 and subpolytope ${\mathcal {B}} \subseteq {\mathcal {A}}$, we obtain a well- defined polytope chain complex

16 $$\begin{aligned} \left( \mathrm {CN}_\bullet \left( \vartheta _a,{\mathcal {A}} |_{{\mathcal {B}}}\right) ,\partial _{\vartheta _a}\right) , \quad \forall a \in {\mathcal {A}}, \end{aligned}$$

satisfying all the bullet points of Theorem 2.14. Any other choice $$\vartheta '=\vartheta '$$$$(\theta ',H',g')$$ does not affect the chain complexes up to chain homotopy equivalence.

Moreover, the inclusion

17 $$\begin{aligned} \iota _{{\mathcal {B}}} :\mathrm {CN}_\bullet (\vartheta _a,{\mathcal {A}}) \longrightarrow \mathrm {CN}_\bullet \left( \vartheta _a,{\mathcal {A}} |_{{\mathcal {B}}}\right) \end{aligned}$$

defines a Novikov-linear chain map for all $a \in {\mathcal {A}}$.

#### Proof

The proof of the first part is literally the same as in Theorems 2.14 and 2.19. To see that the inclusion defines a chain map, it suffices to observe that both boundary operators in (17) are identical upon restricting to the smaller complex $\mathrm {CN}_\bullet (\vartheta _a,{\mathcal {A}})$. $\square$

In the preceding subsection, we also defined a polytope chain complex variant using perturbed sections with respect to non-exact reference pairs (cf. Corollary 2.17). While this variant requires the underlying section to satisfy some a priori smallness conditions, it does agree with the polytope chain complex variant of Theorem 2.14.

#### Theorem 2.22

Let $(\rho ,g)$ and $\theta :{\mathcal {A}} \rightarrow \Omega ^1(M)$ be as in Corollary 2.17, and denote by $\vartheta ^\rho =\vartheta (\theta ,\rho ,g)$ the corresponding perturbed section. Let $\vartheta ^H=\vartheta (\theta ,H,g_H,\varepsilon )$ be any perturbed section as in Theorem 2.14. Then,

18 $$\begin{aligned} \mathrm {CN}_\bullet (\vartheta ^\rho _a,{\mathcal {A}}) \simeq \mathrm {CN}_\bullet (\vartheta ^H_a,{\mathcal {A}}), \quad \forall a \in {\mathcal {A}}. \end{aligned}$$

Let $\theta _i :{\mathcal {A}} \rightarrow \Omega ^1(M)$ be any other two sections $i=0,1$ with reference pairs $(\rho _i,g_i)$ satisfying the conditions of Corollary 2.17. Then, for any two choices $\vartheta ^{\rho _i}=\vartheta (\theta _i,\rho _i,g_i)$, one has

19 $$\begin{aligned} \mathrm {CN}_\bullet (\vartheta ^{\rho _0}_a,{\mathcal {A}}) \simeq \mathrm {CN}_\bullet (\vartheta ^{\rho _1}_a,{\mathcal {A}}), \quad \forall a \in {\mathcal {A}}. \end{aligned}$$

Moreover, both (18) and (19) continue to hold in the restricted case ${\mathcal {B}} \subseteq {\mathcal {A}}$.

#### Proof

The proof idea is again arguing via continuations as in Theorem 2.19 above—we will use the latter as carbon copy and adapt the same notation. Define

$$\begin{aligned} \vartheta ^s=(1-h(s)) \cdot \vartheta ^\rho +h(s) \cdot \vartheta ^H. \end{aligned}$$

By the same logic as in Theorem 2.19, it suffices to control the expression

20 $$\begin{aligned} \int _{\gamma } \vartheta ^s_b-\vartheta ^s_a, \end{aligned}$$

for all $b \in {\mathcal {A}}$, to get the desired continuation chain map to conclude (18). For this purpose, we define

$$\begin{aligned} {\mathcal {S}}^\rho&:=\ \left\{ s \in (-\infty ,0) \, \big | \,\Vert \nabla ^{g_0} \vartheta ^{\rho }_a (\gamma (s)) \Vert _{g_0} \ge \frac{C_\rho }{8} \right\} , \\ {\mathcal {S}}^H&:=\ \left\{ s \in (1,+\infty ) \, \big | \,\Vert \nabla ^{g_1} \vartheta ^H a (\gamma (s)) \Vert _{g_1} \ge \frac{C_H}{\varepsilon \cdot 8} \right\} . \end{aligned}$$

Here, $g_0$ and $g_1$ (abusively) denote Riemannian metrics $g_{\vartheta ^\rho _a}$ and $g_{\vartheta ^H_a}$ coming from Proposition 2.13.

Observe that by assumption and choice of $(\vartheta ^\rho _a,g_0)$, we have

$$\begin{aligned} \Vert \vartheta ^{\rho }_b- \rho \Vert _{g_0} \le 5 \cdot D_0 \cdot \Vert \theta _b- \rho \Vert _g \le \frac{5 \cdot D_0 \cdot C_\rho }{D_0 \cdot 1000}; \end{aligned}$$

see proof of Corollary 2.17 and Proposition 2.13.

Just as in the proof of Theorem 2.19, there is no contribution to (20) for *s* in the complement of $[0,1] \cup {\mathcal {S}}^{\rho } \cup {\mathcal {S}}^H$. At the same time, we can again bound

$$\begin{aligned} \bigg \vert \int _{{\mathcal {S}}^\alpha } \left( \vartheta ^s_b-\vartheta ^s_a \right) \, {\dot{\gamma }}(s) \, \mathrm{d}s \bigg \vert < \frac{1}{10} \cdot E(\gamma ), \end{aligned}$$

and

$$\begin{aligned} \bigg \vert \int _{[0,1]} \left( \vartheta ^s_b-\vartheta ^s_a \right) \, {\dot{\gamma }}(s) \, \mathrm{d}s \bigg \vert \le F \cdot E(\gamma )^{\frac{1}{2}}. \end{aligned}$$

This suffices to obtain the desired control over (20) and proves (18); see proof of Theorem 2.19 for more details. Last but not least, (19) follows by applying (18) twice:

$$\begin{aligned} \mathrm {CN}_\bullet (\vartheta ^{\rho _0}_a,{\mathcal {A}}) \simeq \mathrm {CN}_\bullet (\vartheta ^H_a,{\mathcal {A}}) \simeq \mathrm {CN}_\bullet (\vartheta ^{\rho _1}_a,{\mathcal {A}}), \quad \forall a \in {\mathcal {A}}. \end{aligned}$$

$$\square $$

Theorems 2.19 and 2.22 readily imply:

#### Corollary 2.23

Let $\theta _i :{\mathcal {A}} \rightarrow \Omega ^1(M)$ with $i=0,1$ be two sections and $\vartheta ^i$ associated perturbations as in Theorem 2.14 (or Corollary 2.17). Then, the resulting polytope Novikov homologies are isomorphic

$$\begin{aligned} \mathrm {HN}_\bullet (\vartheta ^0_a,{\mathcal {A}}) \cong \mathrm {HN}_\bullet (\vartheta ^1_a,{\mathcal {A}}), \quad \forall a \in {\mathcal {A}}. \end{aligned}$$

Corollary 2.23 is the analogue to the independence of Morse–Smale pairs $(\alpha ,g)$ in the case of ordinary Novikov homology. The latter can also be recovered from the former by taking the trivial polytope ${\mathcal {A}}=\langle a \rangle$. Nevertheless, keeping track of the section $\theta$, or rather its perturbations, will prove useful, especially when establishing the commutative diagram in the Main Theorem 2.24.

### Non-exact deformations and proof of the main theorem

The power of the polytope machinery will become evident in this subsection—roughly speaking, the notion of polytopes allows us to compare the Novikov homologies coming from two different cohomology classes, see Main Theorem 2.24. In Sect. 3, we present some applications of the Main Theorem 2.24.

#### Theorem 2.24

(Main Theorem) Let ${\mathcal {A}}\subset H^1_{\mathrm {dR}}(M)$ be a polytope and $\theta :{\mathcal {A}} \rightarrow \Omega ^1(M)$ a section. Then, there exists a perturbation $\vartheta :{\mathcal {A}} \rightarrow \Omega ^1(M)$ of $\theta$, such that for every subpolytope ${\mathcal {B}} \subseteq {\mathcal {A}}$ and any two cohomology classes $a, \, b \in {\mathcal {A}}$, there exists a commutative diagram 
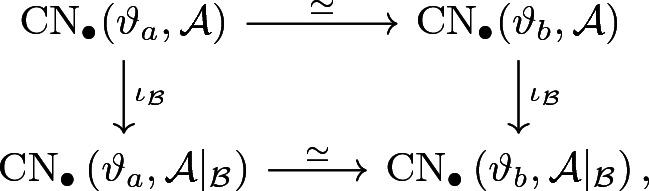


where the horizontal maps are Novikov-linear chain homotopy equivalences. In particular 
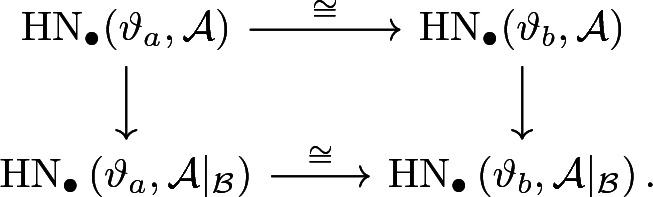
 with all the maps being Novikov linear.

#### Proof

Most of the ideas have already been established in the previous subsection, especially in the proof of Theorems 2.14 and 2.19. Fix a reference Morse–Smale pair (*H*, *g*) and pick a perturbation $\vartheta =\vartheta (\theta ,H,g,\varepsilon )$ as in Theorem 2.14. Let $h :[0,1] \rightarrow {\mathbb {R}}$ be a smooth function as in (13) and define a homotopy

$$\begin{aligned} \vartheta ^s_{ab}:=(1-h(s)) \cdot \vartheta _a+h(s) \cdot \vartheta _b, \quad \forall s \in {\mathbb {R}}. \end{aligned}$$

Pick $g_s$ a smooth homotopy connecting the two metrics $g_{\vartheta _a}$ and $g_{\vartheta _b}$ and assume that $(\vartheta ^s_{ab},g_s)$ is regular (see Remark 2.20). The idea now is to show that the chain continuation map

$$\begin{aligned} \Psi ^{ba} :\mathrm {CN}_\bullet (\vartheta _a,g_{\vartheta _a},{\mathcal {A}}) \longrightarrow \mathrm {CN}_\bullet (\vartheta _b,g_{\vartheta _b},{\mathcal {A}}) \end{aligned}$$

associated with the regular homotopy $(\vartheta ^s_{ab},g_s)$ is well defined and makes the desired diagram commute. The argument that $\Psi ^{ba}$ is a well-defined Novikov chain map is the same as in Theorem 2.19 and follows by controlling terms of the form:

$$\begin{aligned} \int _{\gamma } \vartheta _c-\vartheta ^s_{ab}, \quad \text {with } c \in {\mathcal {A}}. \end{aligned}$$

Note that this time around we do not need to put an *s*-dependence on $\vartheta _c$ (this corresponds to $\vartheta _b$ in the proof of Theorem 2.19), since the endpoints of $\vartheta ^s_{ab}$ have the same zeros as $\vartheta _c$, namely $Z(\vartheta _c)=\mathrm {Crit}(H)$. The *s*-dependence on $\vartheta _c$ is the only bit that used the cohomologous assumption in Theorem 2.19, and indeed, the remaining part of the proof is verbatim the same and is thus omitted.

We use the very same homotopy to define a chain continuation

$$\begin{aligned} \Psi ^{ba} |_{{\mathcal {B}}} :\mathrm {CN}_\bullet \left( \vartheta _a,{\mathcal {A}} |_{{\mathcal {B}}}\right) \longrightarrow \mathrm {CN}_\bullet \left( \vartheta _b,{\mathcal {A}} |_{{\mathcal {B}}} \right) . \end{aligned}$$

From this, we obtain the following commutative diagram on the chain level: 
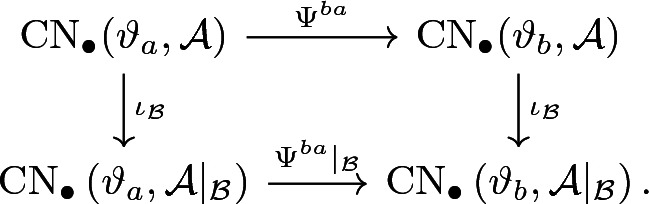
 Here, the $\iota _{\mathcal {B}}$ denote the inclusions (17), which are chain maps (cf. Corollary 2.21). By symmetry and the standard argument, we get continuations $\Psi ^{ab}$ and $\Psi ^{ab} |_{{\mathcal {B}}}$ in the opposite direction by reversing the underlying regular homotopy. It is also easy to see that continuation maps are linear over the underlying Novikov ring. This proves that the two horizontal chain maps above define the desired chain homotopy equivalences. In particular, the chain diagram above induces the desired diagram in homology and thus concludes the proof. $\square$

#### Remark 2.25

In light of Theorems 2.19, 2.22 and Corollary 2.23, one can upgrade Theorem 2.24 and use different sections on the left and on the right of the the diagram, that is 
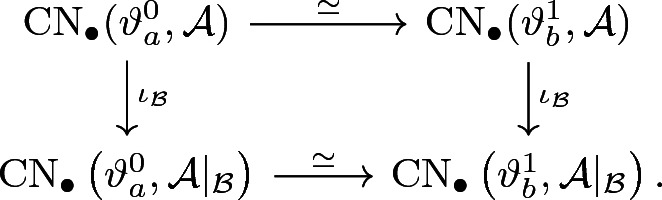
 The upper and lower chain homotopy equivalences, however, do come from compositions of chain continuations rather than genuine chain continuations.

### Twisted Novikov complex

Throughout this subsection, we shall assume that $\theta :{\mathcal {A}} \rightarrow \Omega ^1(M)$ has already been perturbed as in Theorem 2.14.^16^ We present an alternative description of $\mathrm {CN}_\bullet (\theta _a,{\mathcal {A}})$ by means of local coefficients. For an extensive treatment of local coefficients, we recommend [23] and [1, Chapter 2] in the case of Morse homology.

#### Definition 2.26

Let $G \subseteq {\mathbb {R}}$ be an additive subgroup. Then, define

$$\begin{aligned} \mathrm {Nov}(G;{\mathbb {Z}}):=\mathrm {Nov}(G) \end{aligned}$$

as the ring^17^ consisting of formal sums $\sum _{g \in G} n_g t^g$ with $n_g \in {\mathbb {Z}}$, satisfying the finiteness condition

$$\begin{aligned} \forall c \in {\mathbb {R}}:\; \left\{ g \, \big | \, n_g \ne 0, \, g< c \right\} \text { is finite.} \end{aligned}$$

Whenever *G* is the image of a period homomorphism $\Phi _a :\pi _1(M) \rightarrow {\mathbb {R}}$, we write

$$\begin{aligned} \mathrm {Nov}(a):=\mathrm {Nov}(G), \quad G=\mathrm {im}(\Phi _a). \end{aligned}$$

It turns out that $\mathrm {Nov}(a)$ is isomorphic to $\Lambda _a$, where the isomorphism is given by sending a deck transformation $A \in \Gamma _a$ to $t^{\Phi _a(A)}$—both finiteness conditions match and we obtain:

#### Proposition 2.27

For any cohomology class $a \in H^1_{\mathrm {dR}}(M)$, we have

$$\begin{aligned} \Lambda _a \cong \mathrm {Nov}(a), \end{aligned}$$

as rings.

In view of Proposition 2.27, we will also refer to $\mathrm {Nov}(a)$ as Novikov ring of *a*. Inspired by the definition of $\mathrm {Nov}(a)$, we will now define yet another ring $\mathrm {Nov}({\mathcal {A}})$, which will be isomorphic to $\Lambda _{\mathcal {A}}$ almost by definition.

#### Definition 2.28

Let ${\mathcal {A}}:=\langle a_0, \dots , a_k \rangle$ be a polytope. Define

$$\begin{aligned} \mathrm {Nov}({\mathcal {A}};{\mathbb {Z}}):=\mathrm {Nov}({\mathcal {A}}), \end{aligned}$$

as the ring consisting of elements

21 $$\begin{aligned} \sum _{A \in \Gamma _{\mathcal {A}}} n_A \, t_0^{\Phi _{a_0}(A)} \cdots \, t_k^{\Phi _{a_k}(A)}, \quad n_A \in {\mathbb {Z}}, \end{aligned}$$

with a multi-finiteness condition

$$\begin{aligned} \forall l=0,\dots ,k, \, \forall c \in {\mathbb {R}}:\; \left\{ A \, \big | \, n_A \ne 0, \, \Phi _{a_l}(A)< c \right\} \text { is finite.} \end{aligned}$$

Similarly, for any subpolytope ${\mathcal {B}}\subseteq {\mathcal {A}}$, we define a (potentially) larger group

$$\begin{aligned} \mathrm {Nov}\left( {\mathcal {A}} |_{{\mathcal {B}}}\right) \supseteq \mathrm {Nov}({\mathcal {A}}) \end{aligned}$$

consisting of the same formal sums (21), but with a (potentially) less-restrictive multi- finiteness condition

$$\begin{aligned} \text {For all } l \text { such that } a_l \in {\mathcal {B}} \text { and } c \in {\mathbb {R}}:\; \left\{ A \, \big | \, n_A \ne 0, \, \Phi _{a_l}(A) < c \right\} \text { is finite.} \end{aligned}$$

Analogously to Proposition 2.27, we have:^18^

#### Proposition 2.29

For any polytope ${\mathcal {A}}$, we have

$$\begin{aligned} \Lambda _{\mathcal {A}} \cong \mathrm {Nov}({\mathcal {A}}) \end{aligned}$$

as rings. Similarly, we obtain

$$\begin{aligned} \Lambda _{{\mathcal {A}} |_{{\mathcal {B}}}} \cong \mathrm {Nov}({\mathcal {A}} |_{{\mathcal {B}}}), \end{aligned}$$

as well.

Let us mention that these Novikov rings are *commutative* rings, since $\Gamma _{{\mathcal {A}}}$ is abelian. Indeed, the commutator subgroup of $\pi _1(M)$ is contained in every kernel $\ker (a_l)$; in particular

$$\begin{aligned} ABA^{-1}B^{-1} \in \bigcap _{l=0}^k \ker (a_l), \text { thus }ABA^{-1}B^{-1}=0 \in \Gamma _{{\mathcal {A}}}. \end{aligned}$$

Each polytope ${\mathcal {A}}$ comes with a representation

$$\begin{aligned} \rho _{\mathcal {A}} :\pi _1(M,x_0) \times \mathrm {Nov}({\mathcal {A}}) \rightarrow \mathrm {Nov}({\mathcal {A}}), \quad \rho _{{\mathcal {A}}}(\eta ,\lambda )=t_0^{-\Phi _{a_0}(\eta )} \cdots \, t_k^{-\Phi _{a_k}(\eta )} \cdot \lambda . \end{aligned}$$

Sometimes, we will write out $\Phi _{a_l}(\eta )=\int _{\eta } a_l$. The importance of the minus sign will become clear in Definition 2.31. To any such representation, one can associate a *local coefficient system*

$$\begin{aligned} \underline{\mathrm {Nov}}({\mathcal {A}}) :\Pi _1(M) \rightarrow \mathsf {mod}_{\mathrm {Nov}({\mathcal {A}})}, \end{aligned}$$

which is unique up to isomorphisms of local coefficients. We briefly recall the construction:^19^ fix a basepoint $x_0 \in M$ and pick for every $x \in M$ a homotopy class of paths $\{\eta _x\}$ relative to the endpoints $x_0$ and *x*. Set

$$\begin{aligned} \underline{\mathrm {Nov}}({\mathcal {A}})(x):=\mathrm {Nov}({\mathcal {A}}), \quad \forall x \in M. \end{aligned}$$

For every homotopy class $\{\gamma \}$ relative to the endpoints *x* and *y*, we first define a loop

$$\begin{aligned} {\underline{\gamma }}:=\eta _x*\gamma *\eta _y^{-1} :S^1 \rightarrow M, \end{aligned}$$

based at $x_0$, and then define^20^

22 $$\begin{aligned} \underline{\mathrm {Nov}}({\mathcal {A}})(\gamma ) :\underline{\mathrm {Nov}}({\mathcal {A}})(y) \longrightarrow \underline{\mathrm {Nov}}({\mathcal {A}})(x), \underline{\mathrm {Nov}}({\mathcal {A}})(\gamma )(\, \cdot \,) :=\rho _{{\mathcal {A}}} \left( {\underline{\gamma }}, \, \cdot \, \right) .^{20} \end{aligned}$$

Taking a closer look at (22) reveals that the Novikov ring isomorphism $\underline{\mathrm {Nov}}({\mathcal {A}})(\gamma )$ is given by multiplication with

$$\begin{aligned} t_0^{-\int _{{\underline{\gamma }}} a_0} \cdots \, t_k^{-\int _{{\underline{\gamma }}}a_k} \in {\mathbb {Z}}[\Gamma _{{\mathcal {A}}}] \subseteq \mathrm {Nov}({\mathcal {A}}); \end{aligned}$$

hence, we may also view it as a ${\mathbb {Z}}[\Gamma _{{\mathcal {A}}}]$-module isomorphism.

#### Remark 2.30

Usually, local coefficients are considered to take values in the category of abelian groups and are often called “bundle of abelian groups". Mapping into $\mathsf {mod}_{\mathrm {Nov}({\mathcal {A}})}$ will allow us to obtain actual Novikov-module isomorphisms at times where working with bundle of abelian groups would merely grant group isomorphisms.

With this, we define the anticipated twisted Novikov complexes.

#### Definition 2.31

Let $\theta :{\mathcal {A}} \rightarrow \Omega ^1(M)$ be a section as above. We define the **twisted Novikov chain complex groups** by

$$\begin{aligned} \mathrm {CN}_\bullet \left( \theta _a,\underline{\mathrm {Nov}}({\mathcal {A}}) \right) :=\bigoplus _{x \in Z(\theta _a)} \mathrm {Nov}({\mathcal {A}}) \, \langle x \rangle , \quad \forall a \in {\mathcal {A}}. \end{aligned}$$

The **twisted boundary operator** ${\underline{\partial }}={\underline{\partial }}_{\theta _a}$ is defined by

$$\begin{aligned} {\underline{\partial }}(\lambda \, x):= & {} \sum _{y, \, \gamma \in \underline{{\mathcal {M}}}(x,y;\theta _a)} \underline{\mathrm {Nov}}({\mathcal {A}})(\gamma ^{-1})(\lambda ) \, y\\= & {} \sum _{y, \, \gamma \in \underline{{\mathcal {M}}}(x,y;\theta _a)} t_0^{\int _{{\underline{\gamma }}} a_0} \, \cdots \; t_k^{\int _{{\underline{\gamma }}} a_k} \cdot \lambda \, y, \quad \forall \lambda \in \mathrm {Nov}({\mathcal {A}}). \end{aligned}$$

The twisted chain complexes

$$\begin{aligned} \left( \mathrm {CN}_\bullet \left( \theta _a,\underline{\mathrm {Nov}}\left( {\mathcal {A}} |_{{\mathcal {B}}}\right) \right) , {\underline{\partial }}_{\theta _a} \right) , \quad \forall a \in {\mathcal {A}} \end{aligned}$$

are defined analogously. The corresponding **twisted Novikov homologies** are denoted by

$$\begin{aligned} \mathrm {HN}_\bullet \left( \theta _a,\underline{\mathrm {Nov}}({\mathcal {A}}) \right) \text { and } \mathrm {HN}_\bullet \left( \theta _a,\underline{\mathrm {Nov}}\left( {\mathcal {A}} |_{{\mathcal {B}}}\right) \right) , \quad \forall a \in {\mathcal {A}}, \, {\mathcal {B}} \subseteq {\mathcal {A}}. \end{aligned}$$

For every flow line $\gamma \in \underline{{\mathcal {M}}}(x,y;\theta _a)$, we call

$$\begin{aligned} \underline{\mathrm {Nov}}({\mathcal {A}})(\gamma ^{-1})=t_0^{\int _{{\underline{\gamma }}} a_0}\cdots t_k^{\int _{{\underline{\gamma }}} a_k} \end{aligned}$$

the **Novikov twist** of $\gamma$.

#### Remark 2.32

A priori it is not clear that ${\underline{\partial }}$ maps into the prescribed chain complex. We will prove this in the next subsection; see Proposition 2.34.

The Novikov twist of $\gamma$ determines the lifting behavior of $\gamma$. Indeed, if $\gamma _0, \gamma _1$ are two paths from *x* to *y* with unique lifts ${\tilde{\gamma }}_1(0)={\tilde{\gamma }}_0(0)={\tilde{x}}$, then

$$\begin{aligned} {\tilde{\gamma }}_0(1)={\tilde{\gamma }}_1(1)&\iff \int _{\gamma _0*\gamma _1^{-1}} a_l=0, \, \forall l=0,\dots ,k \\&\iff \int _{\gamma _0}a_l =\int _{\gamma _1}a_l, \, \forall l=0,\dots ,k \\&\iff \int _{\underline{\gamma _0}}a_l =\int _{\underline{\gamma _1}}a_l, \, \forall l=0,\dots ,k \\&\iff t_0^{\int _{\underline{\gamma _0}} a_0} \cdots \, t_k^{\int _{\underline{\gamma _0}}a_k}=t_0^{\int _{\underline{\gamma _1}} a_0} \cdots \, t_k^{\int _{\underline{\gamma _1}}a_k}. \end{aligned}$$

This will be key in the next subsection.

As the next examples show, we recover the *twisted Morse chain complex* and its homology as a special case.

#### Example 2.33

Pick ${\mathcal {A}}=\langle 0, a \rangle$ and a section $\theta :{\mathcal {A}} \rightarrow \Omega ^1(M)$. Then

$$\begin{aligned} \mathrm {Nov}({\mathcal {A}}|_a) \cong \mathrm {Nov}(a) \cong \Lambda _a, \end{aligned}$$

thanks to Proposition 2.27 and Example 2.3. Furthermore, the twisted chain complex

$$\begin{aligned} \mathrm {CN}_\bullet \left( \theta _0,\underline{\mathrm {Nov}}({\mathcal {A}}|_a)\right) =\mathrm {CN}_\bullet \left( \theta _0,\underline{\mathrm {Nov}}(a)\right) \end{aligned}$$

agrees with the twisted Morse complex

$$\begin{aligned} \mathrm {CM}_\bullet \left( h, \underline{\mathrm {Nov}}(a) \right) , \text { where } \theta _0=dh. \end{aligned}$$

### Comparing twisted and polytope complexes

In general, the same issues as in Sect. 2.2 arises when trying to prove that the twisted Novikov complexes are well defined: it is not clear whether ${\underline{\partial }}$ maps into the desired chain complex. This is a non-issue in the special case of $\theta _0=dh$, i.e., twisted Morse homology—the reason is that the 0-dimensional moduli spaces $\underline{{\mathcal {M}}}(x,y;h)$ are compact, hence finite. Compare this to [20]. In the following however, we will see that the twisted chain *groups* can always be identified with $\mathrm {CN}_\bullet (\theta _a,{\mathcal {A}})$, so that ${\underline{\partial }}$ and $\partial$ agree, which then resolves the well-definedness issue by Theorem 2.14. In other words, the twisted chain complex is an equivalent description of the polytope chain complex.

#### Proposition 2.34

Let $\theta :{\mathcal {A}} \rightarrow \Omega ^1(M)$ be a section as above. Then, the twisted and polytope Novikov chain groups are isomorphic

23 $$\begin{aligned} \mathrm {CN}_\bullet \left( \theta _a,\underline{\mathrm {Nov}}({\mathcal {A}}) \right) \longleftrightarrow \mathrm {CN}_\bullet (\theta _a,{\mathcal {A}}), \end{aligned}$$

as Novikov modules. The isomorphism is preserved upon restrictions ${\mathcal {A}} |_{{\mathcal {B}}}$, and $\partial = {\underline{\partial }}$ up to the identification (23).

In particular, the twisted Novikov chain complexes are well defined with

$$\begin{aligned} \mathrm {HN}_\bullet \left( \theta _a,\underline{\mathrm {Nov}}({\mathcal {A}}) \right) =\mathrm {HN}_\bullet (\theta _a,{\mathcal {A}}) \text { and } \mathrm {HN}_\bullet \left( \theta _a,\underline{\mathrm {Nov}}\left( {\mathcal {A}} |_{{\mathcal {B}}}\right) \right) =\mathrm {HN}_\bullet \left( \theta _a,{\mathcal {A}} |_{{\mathcal {B}}}\right) , \end{aligned}$$

for all $a \in {\mathcal {A}}$ and subpolytopes ${\mathcal {B}} \subseteq {\mathcal {A}}$.

#### Proof

First of all recall that we can view the *i*-th polytope Novikov chain groups as finitely generated Novikov modules by fixing a finite set of preferred lifts ${\tilde{x}}_m \in \pi ^{-1}(x_m)$, for each zero $x_m$ of $\theta _a$ of index *i*

$$\begin{aligned} \mathrm {CN}_i(\theta _a,{\mathcal {A}}) \cong \bigoplus _m \Lambda _{\mathcal {A}} \langle {\tilde{x}}_m \rangle , \quad \text { as Novikov ring modules}. \end{aligned}$$

Since $\Lambda _{\mathcal {A}} \cong \mathrm {Nov}({\mathcal {A}})$ (cf. Proposition 2.29), we end up with

$$\begin{aligned} \mathrm {CN}_i(\theta _a,{\mathcal {A}}) \cong \bigoplus _m \Lambda _{\mathcal {A}} \langle {\tilde{x}}_m \rangle \cong \bigoplus _m \mathrm {Nov}({\mathcal {A}}) \langle x_m \rangle =\mathrm {CN}_i\left( \theta _a,\underline{\mathrm {Nov}}({\mathcal {A}})\right) . \end{aligned}$$

Both boundary operators $\partial$ and ${\underline{\partial }}$ are $\Lambda _{\mathcal {A}}$- and $\mathrm {Nov}({\mathcal {A}})$-linear, and thus, it suffices to compare $\partial {\tilde{x}}_m$ and ${\underline{\partial }} x_m$. On the one hand, we have

24 $$\begin{aligned} \partial {\tilde{x}}_m=\sum _n \lambda _{m,n} {\tilde{y}}_n, \text { with } \lambda _{m,n}=\sum _{A \in \Gamma _{\mathcal {A}}}\#_{\mathrm {alg}} \, \underline{{\mathcal {M}}}\left( {\tilde{x}}_m,A {\tilde{y}}_n;{\tilde{f}}_{\theta _a}\right) \, A \in \Lambda _{{\mathcal {A}}},\nonumber \\ \end{aligned}$$

and on the other hand

25 $$\begin{aligned} {\underline{\partial }} x_m=\sum _n \left( \sum _{\gamma \in \underline{{\mathcal {M}}}\left( x_m,y_n,\theta _a \right) } t_0^{\int _{{\underline{\gamma }}} a_0 } \, \cdots \; t_k^{\int _{{\underline{\gamma }}} a_k} \right) \, y_n. \end{aligned}$$

In the previous subsection, we have seen that the Novikov twist $t_0^{\int _{{\underline{\gamma }}} a_0} \, \cdots \; t_k^{\int _{{\underline{\gamma }}} a_k}$ of $\gamma$ uniquely determines the lifting behavior of $\gamma$. Thus, if ${\tilde{\gamma }}$ denotes the unique lift which starts at ${\tilde{x}}_n$ and ends at some ${\tilde{y}}$, we get

$$\begin{aligned} {\tilde{y}}=A{\tilde{y}}_n, \end{aligned}$$

with $A \in \Gamma _{\mathcal {A}} \subset \Lambda _{\mathcal {A}}$ corresponding to $t_0^{\int _{{\underline{\gamma }}} a_0} \, \cdots \; t_k^{\int _{{\underline{\gamma }}}a_k} \in \mathrm {Nov}({\mathcal {A}})$. This proves that (24) and (25) agree up to identifying the respective isomorphic Novikov rings. The same proof also shows that the restricted complexes associated with ${\mathcal {A}} |_{{\mathcal {B}}}$ agree.

With the identification of twisted and polytope complexes at hand, we can invoke Theorem 2.14 (recall that we already assumed that $\theta$ is perturbed accordingly) and deduce that the twisted chain complex is well defined. By the first part, it follows that the corresponding homologies agree. This finishes the proof. $\square$

#### Remark 2.35

The twisted complex can be used to deduce properties of the polytope complex and vice versa. For instance, trying to prove that $\partial ^2=0$ is equivalent to proving ${\underline{\partial }}^2=0$, which has a far more pleasant proof—the reason is that the Novikov twists are nicely behaved with respect to the compactification of the moduli spaces, see for instance [20, Proposition 1].

## Applications of the main theorem

### The 0-vertex trick and the Morse–Eilenberg Theorem

All the applications we are about to present boil down to what we call the *0-vertex trick*. The idea is to relate the polytope chain groups to twisted Morse chain groups by extending the underlying polytope ${\mathcal {A}}$ with $0 \in H^1_{\mathrm {dR}}(M)$ as an additional vertex, and then using the Main Theorem 2.24. This trick suffices to prove the (twisted) Novikov Morse Homology Theorem (cf. Theorem 3.3 and Corollary 3.4).

For the polytope Novikov Principle (cf. Theorem 3.6), we shall need a Morse variant of the Eilenberg Theorem [3, Theorem 24.1], which has been proven in [1, Theorem 2.21]. We will state and prove a slightly stronger version in the context of Novikov theory down below; see Lemma 3.2.

#### Lemma 3.1

(0-vertex trick) Let $\theta :{\mathcal {A}} \rightarrow \Omega ^1(M)$ be a section, ${\mathcal {B}} \subseteq {\mathcal {A}}$ a subpolytope, and ${\mathcal {A}}^0$ the polytope spanned by the vertices of ${\mathcal {A}}$ and 0.^21^ Let $\theta ^0 :{\mathcal {A}}^0 \rightarrow \Omega ^1(M)$ be a section extending $\theta$. Then

$$\begin{aligned} {\mathbb {Z}}[\Gamma _{{\mathcal {A}}}]={\mathbb {Z}}[\Gamma _{{\mathcal {A}}^0}] \quad \text {and}\quad \underline{\mathrm {Nov}}({\mathcal {A}} |_{{\mathcal {B}}}) = \underline{\mathrm {Nov}}({\mathcal {A}}^0 |_{{\mathcal {B}}}). \end{aligned}$$

In particular, there exists a perturbed section $\vartheta ^0 :{\mathcal {A}}^0 \rightarrow \Omega ^1(M)$, such that

$$\begin{aligned} \mathrm {CN}_\bullet \left( \vartheta ^0_a,{\mathcal {A}} |_{{\mathcal {B}}}\right) =\mathrm {CN}_\bullet \left( \vartheta ^0_a,{\mathcal {A}}^0 |_{{\mathcal {B}}}\right) \simeq \mathrm {CN}_\bullet \left( \vartheta ^0_0,{\mathcal {A}}^0 |_{{\mathcal {B}}} \right) =\mathrm {CM}_\bullet \left( h,\underline{\mathrm {Nov}}({\mathcal {A}} |_{{\mathcal {B}}}) \right) , \quad \forall a \in {\mathcal {A}}, \end{aligned}$$

as chain complexes, where $dh=\vartheta ^0_0$.

#### Proof

Since $\ker (0)=\pi _1(M)$, adding 0 as vertex does not affect the underlying abelian cover, i.e., ${\widetilde{M}}_{\mathcal {A}}={\widetilde{M}}_{{\mathcal {A}}^0}$, also see Example 2.3. Thus, the deck transformation groups $\Gamma _{{\mathcal {A}}}$ and $\Gamma _{{\mathcal {A}}^0}$ are equal and so are the respective group rings. The finiteness conditions for both $\mathrm {Nov}({\mathcal {A}} |_{{\mathcal {B}}})$ and $\mathrm {Nov}({\mathcal {A}}^0 |_{{\mathcal {B}}})$ are determined by the subpolytope ${\mathcal {B}} \subseteq {\mathcal {A}}$, and thus, by the group ring equality, we also deduce

$$\begin{aligned} \mathrm {Nov}({\mathcal {A}} |_{{\mathcal {B}}})=\mathrm {Nov}({\mathcal {A}}^0 |_{{\mathcal {B}}}). \end{aligned}$$

The equality as local coefficient systems then also follows by observing that the period homomorphism $\Phi _0$ is identically zero, and hence, $t^{\Phi _0(A)}=1$ for all $A \in \Gamma _{{\mathcal {A}}^0}$. Pick $\vartheta ^0$ as in Theorem 2.14 (or Corollary 2.17). The first chain polytope equality follows from the Novikov rings being equal and the chain homotopy equivalence stems from the Main Theorem 2.24. The last equality follows from Example 2.33 and the equality of local coefficient systems above.

$$\square $$

We conclude the subsection by stating and proving a chain-level Morse variant of the Eilenberg Theorem [1, Theorem 2.21]

#### Lemma 3.2

(Morse–Eilenberg Theorem) Let $h :M \rightarrow {\mathbb {R}}$ be Morse function, ${\mathcal {A}}$ a polytope, and ${\mathcal {B}} \subseteq {\mathcal {A}}$ a subpolytope. Then

$$\begin{aligned} \mathrm {CM}_\bullet \left( {\tilde{h}}\right) \otimes _{{\mathbb {Z}}[\Gamma _{{\mathcal {A}}}]} \mathrm {Nov}({\mathcal {A}} |_{{\mathcal {B}}}) \cong \mathrm {CM}_\bullet \left( h,\underline{\mathrm {Nov}}({\mathcal {A}} |_{{\mathcal {B}}}) \right) \end{aligned}$$

as chain complexes over $\mathrm {Nov}({\mathcal {A}} |_{{\mathcal {B}}})$, where ${\tilde{h}}=h\circ \pi$.

The proof of [1, Theorem 2.21] constructs a group chain isomorphism $\Psi$ and a careful inspection reveals that $\Psi$ defines a Novikov-module chain isomorphism when working with the according local coefficient system $\underline{\mathrm {Nov}}({\mathcal {A}} |_{{\mathcal {B}}})$. Nevertheless, we decided to give a full proof of Lemma 3.2 using the tools developed in the previous sections.

#### Proof of Lemma 3.2

First of all, observe that $\mathrm {CM}_\bullet \left( {\tilde{h}}\right)$ is a finite ${\mathbb {Z}}[\Gamma _{\mathcal {A}}]$-module

$$\begin{aligned} \mathrm {CM}_\bullet ({\tilde{h}})=\bigoplus _m {\mathbb {Z}}[\Gamma _{\mathcal {A}}] \, \langle {\tilde{x}}_m \rangle . \end{aligned}$$

Here, $\{{\tilde{x}}_m\}$ denotes a finite set of preferred lifts as in the proof of Proposition 2.34. Define

$$\begin{aligned} \Psi :\mathrm {CM}_\bullet \left( {\tilde{h}}\right) \otimes _{{\mathbb {Z}}[\Gamma _{{\mathcal {A}}}]} \mathrm {Nov}({\mathcal {A}} |_{{\mathcal {B}}}) \longrightarrow \mathrm {CM}_\bullet \left( h,\underline{\mathrm {Nov}}({\mathcal {A}} |_{{\mathcal {B}}}) \right) , \; \Psi ({\tilde{x}}_m \otimes \lambda )=\lambda \, \langle x_m \rangle . \end{aligned}$$

By the above observation, $\Psi$ is a well defined ${\mathbb {Z}}[\Gamma _{\mathcal {A}}]$-linear map. It is clear that $\Psi$ is surjective. For injectivity, we observe that

$$\begin{aligned} \Psi ({\tilde{x}}_m \otimes \lambda )=\Psi ({\tilde{x}}_n \otimes \mu ) \iff \lambda \, \langle x_m \rangle = \mu \, \langle x_n \rangle . \end{aligned}$$

Therefore, we must have $\lambda =\mu$ and $x_m=x_n$, and hence, ${\tilde{x}}_m={\tilde{x}}_n$—recall that we are working with a preferred set of critical points in each fiber. This proves injectivity.

Next, we show that $\Psi$ is $\mathrm {Nov}({\mathcal {A}} |_{{\mathcal {B}}})$-linear.^22^ Pick $\lambda , \, \mu \in \mathrm {Nov}({\mathcal {A}} |_{{\mathcal {B}}})$ and observe

$$\begin{aligned} \Psi (\mu \cdot ({\tilde{x}}_m \otimes \lambda ))=\Psi ({\tilde{x}}_m \otimes (\mu \cdot \lambda ))=(\mu \cdot \lambda ) \, \langle x_m \rangle =\mu \cdot (\lambda \, \langle x_m \rangle )=\mu \cdot \Psi ({\tilde{x}}_m \otimes \lambda ). \end{aligned}$$

Hence, $\Psi$ is a Novikov-linear isomorphism. We are only left to show ${\underline{\partial }} \circ \Psi =\Psi \circ (\partial ^M \otimes \mathrm {id})$. Recall that $\partial ^M$ is defined by counting Novikov–Morse trajectories of ${\tilde{h}}$ on ${\widetilde{M}}_{\mathcal {A}}$. In particular, the boundary operator $\partial$ on $\mathrm {CN}_\bullet \left( dh,{\mathcal {A}}|_{{\mathcal {B}}}\right)$ agrees with $\partial ^M$ on $\mathrm {Crit}({\tilde{h}})$. Let us adopt the notation of the proof of Proposition 2.34 and write

$$\begin{aligned} \partial ^M {\tilde{x}}_m=\partial {\tilde{x}}_m= \sum _n \lambda _{m,n} \, {\tilde{y}}_n, \quad \lambda _{m,n} \in {\mathbb {Z}}[\Gamma _{{\mathcal {A}}}].^{23} \end{aligned}$$

^23^ Therefore

$$\begin{aligned} \Psi \circ (\partial ^M \otimes \mathrm {id}) \, {\tilde{x}}_m \otimes \lambda =\Psi \bigg ( \sum _n \lambda _{m,n} \, {\tilde{y}}_n \otimes \lambda \bigg )= \sum _n \lambda _{m,n} \cdot \lambda \, \langle y_n \rangle , \end{aligned}$$

by Novikov linearity of $\Psi$. On the other hand, Proposition 2.34 says that up to identifying ${\tilde{x}}_m$ and $x_m$, we have $\partial {\tilde{x}}_m = {\underline{\partial }} \, x_m$, and thus

$$\begin{aligned} {\underline{\partial }} \circ \Psi ({\tilde{x}}_m \otimes \lambda )={\underline{\partial }}(\lambda \, \langle x_m \rangle )=\lambda \, {\underline{\partial }} \, x_m= \lambda \cdot \sum _n \lambda _{m,n} \, \langle y_n \rangle . \end{aligned}$$

However, $\mathrm {Nov}({\mathcal {A}} |_{{\mathcal {B}}})$ is a commutative ring, and hence, we conclude the chain property of $\Psi$ and thus that $\Psi$ defines a Novikov-linear chain isomorphism.

$$\square $$

### The twisted Novikov Morse Homology Theorem

Using the results developed in Sect. 2 and the 0-vertex trick (cf. Lemma 3.1), we are going to prove:

#### Theorem 3.3

Let *f* be a Morse function and $a \in H^1_{\mathrm {dR}}(M)$ a cohomology class. Then, for every Morse representative $\alpha \in a$, there exists a chain homotopy equivalence

$$\begin{aligned} \mathrm {CN}_\bullet (\alpha ) \simeq \mathrm {CM}_\bullet \left( f, \underline{\mathrm {Nov}}(a) \right) \end{aligned}$$

of Novikov modules.

One can prove that the twisted Morse homology computes singular homology with coefficients $\underline{\mathrm {Nov}}(a)$; see [1, Theorem 4.1].^24^ Combining this with Theorem 3.3 and taking the homology then shows:

#### Corollary 3.4

(Twisted Novikov Homology Theorem) For any cohomology class $a \in H^1_{\mathrm {dR}}(M)$, there exists an isomorphism

$$\begin{aligned} \mathrm {HN}_\bullet (a) \cong \mathrm {H}_\bullet (M,\underline{\mathrm {Nov}}(a)) \end{aligned}$$

of Novikov modules.

#### Remark 3.5

This is a slightly different incarnation of the classical Novikov Morse Homology Theorem as Corollary 3.4 relates the Novikov homology to twisted singular homology rather than equivariant singular homology. Moreover, as the proof will show, we do *not* invoke the Eilenberg Theorem (or its Morse analogue from the previous subsection) and instead produce a direct connection between the Novikov complex and the twisted Morse complex via the 0-vertex trick—this chain of arguments appears to be novel.

#### Proof of Theorem 3.3

Pick $0, \,a$ in $H^1_{\mathrm {dR}}(M)$, set ${\mathcal {A}}:=\langle 0,a \rangle$, and consider any section

$$\begin{aligned} \theta :{\mathcal {A}} \rightarrow \Omega ^1(M). \end{aligned}$$

Up to perturbing $\theta$, we may assume that $\theta$ is a section $\vartheta ^0$ (note that here ${\mathcal {A}}^0={\mathcal {A}}$) as in Lemma 3.1. In particular, setting ${\mathcal {B}}=\langle a \rangle$ and invoking Lemma 3.1, we get a Novikov chain homotopy equivalence

$$\begin{aligned} \mathrm {CN}_\bullet \left( \theta _a,{\mathcal {A}} |_a \right) \simeq \mathrm {CM}_\bullet \left( h, \underline{\mathrm {Nov}}({\mathcal {A}} |_a) \right) . \end{aligned}$$

From Example 2.33, we deduce that $\underline{\mathrm {Nov}}\left( {\mathcal {A}} |_a \right) =\underline{\mathrm {Nov}}(a)$, and therefore, we obtain

$$\begin{aligned} \mathrm {CN}_\bullet (\theta _a) \simeq \mathrm {CM}_\bullet (h,\underline{\mathrm {Nov}}(a)) \end{aligned}$$

as Novikov modules. Twisted Morse homology, just as ordinary Morse homology, does not depend on the choice of Morse function. Indeed, using continuation methods, one can prove

$$\begin{aligned} \mathrm {CN}_\bullet (h,\underline{\mathrm {Nov}}(a)) \simeq \mathrm {CM}_\bullet (f,\underline{\mathrm {Nov}}(a)) \end{aligned}$$

as Novikov modules.^25^ This proves

$$\begin{aligned} \mathrm {CN}_\bullet (\theta _a) \simeq \mathrm {CM}_\bullet (f,\underline{\mathrm {Nov}}(a)) \end{aligned}$$

as Novikov modules. Observing $\theta _a \in a$ and taking the homology on both sides complete the proof. $\square$

### A polytope Novikov principle

Combining the two lemmata from Sect. 3.1 and the Main Theorem 2.24, we will prove a *polytope Novikov Principle* (see Theorem 3.6). As a corollary, we recover the ordinary Novikov Principle (cf. Corollary 3.8). In fact, the results here also cover those in Sect. 3.2. We opted to keep them apart to emphasize the novelty of the “twisted" approach to the Novikov Homology Theorem (see Remark 3.5).

The polytope Novikov Principle yields a new proof (in the abelian case) to a recent result due to Pajitnov [17, Theorem 5.1].

#### Theorem 3.6

(Polytope Novikov Principle) Let $\theta :{\mathcal {A}} \rightarrow \Omega ^1(M)$ be any section and ${\mathcal {B}}\subseteq {\mathcal {A}}$ a subpolytope. Then, there exists a perturbed section $\vartheta :{\mathcal {A}} \rightarrow \Omega ^1(M)$, such that

26 $$\begin{aligned} \mathrm {CN}_\bullet \left( \vartheta _a,{\mathcal {A}} |_{{\mathcal {B}}} \right) \simeq C_\bullet \left( {\widetilde{M}}_{\mathcal {A}}\right) \otimes _{{\mathbb {Z}}[\Gamma _{\mathcal {A}}]}\mathrm {Nov}({\mathcal {A}}|_{\mathcal {B}}), \quad \forall a \in {\mathcal {A}}, \end{aligned}$$

as Novikov modules.

Here, $C_\bullet$ denotes the singular chain complex with ${\mathbb {Z}}$-coefficients.

#### Proof of Theorem 3.6

Let $\theta ^0 :{\mathcal {A}}^0 \rightarrow \Omega ^1(M)$ be a section that extends $\theta$ with ${\mathcal {A}}^0$ the polytope generated by the vertices of ${\mathcal {A}}$ and 0. By the 0-vertex trick, i.e., Lemma 3.1, there exists a perturbed section $\vartheta ^0 :{\mathcal {A}}^0 \rightarrow \Omega ^1(M)$, such that

$$\begin{aligned} \mathrm {CN}_\bullet \left( \vartheta ^0_a,{\mathcal {A}} |_{{\mathcal {B}}}\right) \simeq \mathrm {CM}_\bullet \left( h,\underline{\mathrm {Nov}}({\mathcal {A}} |_{{\mathcal {B}}}) \right) , \quad \forall a \in {\mathcal {A}} \end{aligned}$$

as Novikov modules. Combining this with Lemma 3.2, we obtain

$$\begin{aligned} \mathrm {CN}_\bullet \left( \vartheta ^0_a,{\mathcal {A}} |_{{\mathcal {B}}} \right) \simeq \mathrm {CM}_\bullet \left( {\tilde{h}} \right) \otimes _{{\mathbb {Z}}[\Gamma _{\mathcal {A}}]} \mathrm {Nov}({\mathcal {A}} |_{{\mathcal {B}}}), \quad \forall a \in {\mathcal {A}} \end{aligned}$$

as Novikov modules. From standard Morse theory, we know that the Morse chain complex $\mathrm {CM}_\bullet \left( {\tilde{h}} \right)$ is chain homotopy equivalent over ${\mathbb {Z}}[\Gamma _{{\mathcal {A}}}]$ to the singular chain complex $C_\bullet \left( {\widetilde{M}}_{\mathcal {A}} \right)$; see for instance [16, Page 415] and [15, Appendix]. Denote by

$$\begin{aligned} i :\mathrm {CM}_\bullet \left( {\tilde{h}} \right) \longrightarrow C_\bullet \left( {\widetilde{M}}_{\mathcal {A}} \right) , \; j :C_\bullet \left( {\widetilde{M}}_{\mathcal {A}} \right) \longrightarrow \mathrm {CM}_\bullet \left( {\tilde{h}} \right) \end{aligned}$$

such a chain homotopy equivalence. Then, one can easily check that $i \otimes \mathrm {id}_{\mathrm {Nov}({\mathcal {A}} |_{\mathcal {B}})}$ and $j \otimes \mathrm {id}_{\mathrm {Nov}({\mathcal {A}} |_{\mathcal {B}})}$ define a Novikov-linear chain homotopy equivalence

$$\begin{aligned} \mathrm {CM}_\bullet \left( {\tilde{h}} \right) \otimes _{{\mathbb {Z}}[\Gamma _{{\mathcal {A}}}]} \mathrm {Nov}({\mathcal {A}} |_{{\mathcal {B}}}) \simeq C_\bullet \left( {\widetilde{M}}_{\mathcal {A}} \right) \otimes _{{\mathbb {Z}}[\Gamma _{{\mathcal {A}}}]} \mathrm {Nov}({\mathcal {A}} |_{{\mathcal {B}}}).^{26} \end{aligned}$$

^26^ Hence

$$\begin{aligned} \mathrm {CN}_\bullet \left( \vartheta ^0_a,{\mathcal {A}} |_{{\mathcal {B}}} \right) \simeq \mathrm {CM}_\bullet \left( {\tilde{h}} \right) \otimes _{{\mathbb {Z}}[\Gamma _{\mathcal {A}}]} \mathrm {Nov}({\mathcal {A}} |_{{\mathcal {B}}}) \simeq C_\bullet \left( {\widetilde{M}}_{\mathcal {A}} \right) \otimes _{{\mathbb {Z}}[\Gamma _{{\mathcal {A}}}]} \mathrm {Nov}({\mathcal {A}} |_{{\mathcal {B}}}), \quad \forall a \in {\mathcal {A}}. \end{aligned}$$

Setting $\vartheta :=\vartheta ^0 |_{{\mathcal {A}}}$ finishes the proof. $\square$

#### Remark 3.7

If one is interested in a particular Morse form $\omega$, then the following improvement can be made: let $(\omega ,g)$ be Morse–Smale and assume that ${\mathcal {A}}$ is a polytope around $[\omega ]$ that admits a section $\theta :{\mathcal {A}} \rightarrow \Omega ^1(M)$ sufficiently close to $(\omega ,g)$ in the sense of Corollary 2.17. Denote by $\vartheta ^\omega :{\mathcal {A}} \rightarrow \Omega ^1(M)$ the associated perturbation with reference pair $(\omega ,g)$. The section $\vartheta =\vartheta ^0 |_{{\mathcal {A}}}$ in the proof above might come from an exact reference pair, since 0 could a priori be far away from $\theta$. However, Corollary 2.23 asserts

$$\begin{aligned} \mathrm {CN}(\vartheta ^{\omega }_a,{\mathcal {A}} |_{{\mathcal {B}}}) \simeq \mathrm {CN}(\vartheta _a,{\mathcal {A}} |_{{\mathcal {B}}}), \quad \forall a \in {\mathcal {A}}. \end{aligned}$$

Combining this with (26) for $a=[\omega ]$ gives

$$\begin{aligned} \mathrm {CN}_\bullet (\omega ,{\mathcal {A}}|_{{\mathcal {B}}}) \simeq C_\bullet \left( {\widetilde{M}}_{\mathcal {A}}\right) \otimes _{{\mathbb {Z}}[\Gamma _{{\mathcal {A}}}]} \mathrm {Nov}({\mathcal {A}} |_{{\mathcal {B}}}), \end{aligned}$$

since $$\vartheta ^\omega _{[\omega ]}=\omega $$.

In the special case of ordinary Novikov theory, i.e., ${\mathcal {A}}=\langle a \rangle$ and ${\mathcal {A}}^0=\langle 0,a\rangle$, Theorem 3.6 reduces to the ordinary Novikov Principle.

#### Corollary 3.8

(Ordinary Novikov Principle) Let $(\alpha ,g)$ be Morse–Smale. Then

$$\begin{aligned} \mathrm {CN}_\bullet (\alpha ) \simeq C_\bullet \left( {\widetilde{M}}_a\right) \otimes _{{\mathbb {Z}}[\Gamma _a]} \mathrm {Nov}(a). \end{aligned}$$

#### Proof

Set $a=[\alpha ]$, pick ${\mathcal {A}}=\langle a \rangle$, ${\mathcal {B}}={\mathcal {A}}$ and $\theta :{\mathcal {A}} \rightarrow \Omega ^1(M)$ the smooth section defined by $\theta _a=\alpha$. The section $\theta$ is obviously close to $(\alpha ,g)$, and thus, Theorem 3.6 and Remark 3.7 imply

$$\begin{aligned} \mathrm {CN}_\bullet (\alpha ) \simeq C_\bullet \left( {\widetilde{M}}_a \right) \otimes _{{\mathbb {Z}}[\Gamma _a]} \mathrm {Nov}(a) \end{aligned}$$

as Novikov modules. $\square$

Even though it is hidden in the proof above, the main idea is still to use perturbations $\vartheta ^0 :{\mathcal {A}}^0 \rightarrow \Omega ^1(M)$ associated with an exact reference pair (*H*, *g*). Recall that these sections $\vartheta ^0$ are constructed by a “shift-and-scale" procedure, so that each $\vartheta ^0_a$ is dominated by the exact term $\frac{1}{\varepsilon } dH$. This strategy to recover the ordinary Novikov Principle has been known among experts for quite awhile; see [14, Page 302] for a historical account, [13, Page 548] and [9, Theorem 3.5.2]. However, our approach is slightly different as it does not make use of gradient like vector fields.

We conclude the present subsection by explaining how to recover [17, Theorem 5.1] from the polytope Novikov principle. For the reader’s convenience, we briefly recall Pajitnov’s setting, keeping the notation as close as possible to [17]. Fix a Morse–Smale pair $(\omega ,g)$ on *M* and let $p :{\widehat{M}} \rightarrow M$ be a regular cover, such that $p^*[\omega ]=0$. Denote by *r* the rank of $\omega$^27^ and define $G=\mathrm {Deck}({\widehat{M}})$. Viewing the period homomorphism $\Phi _\omega$ on $H_1(M;{\mathbb {Z}})$, we get a splitting

$$\begin{aligned} H_1(M;{\mathbb {Z}}) \cong {\mathbb {Z}}^r \oplus \ker [\omega ]. \end{aligned}$$

Pajitnov calls a family of homomorphism

$$\begin{aligned} \Psi _1,\dots , \Psi _r :{\mathbb {Z}}^r \rightarrow {\mathbb {Z}}\end{aligned}$$

a $\Phi _\omega$-regular family if

- the $\Psi _i$ span $\hom _{\mathbb {Z}}({\mathbb {Z}}^r,{\mathbb {Z}})$ and
- the coordinates of $\Phi _\omega :{\mathbb {Z}}^r \rightarrow {\mathbb {R}}$ in the basis $\Psi _i$ are strictly positive.

We shall call $\Psi =\lbrace \Psi _i \rbrace$ a $\Phi _\omega$-*semi-regular* family whenever the first bullet point above is satisfied. One should not be fooled by the length of name—the existence of a semi-regular family is obvious and merely an algebraic statement.

To every (semi)-regular family $\Psi =\{\Psi _1,\dots ,\Psi _r\}$, we associate the conical Novikov ring

$$\begin{aligned} {\widehat{\Lambda }}_\Psi =\bigcap _{i=1}^r {\widehat{{\mathbb {Z}}}}[G]^{\Psi _i}. \end{aligned}$$

The conical Novikov chain complex $({\mathcal {N}}_\bullet (\omega ),\partial )$ is defined as

$$\begin{aligned} {\mathcal {N}}_i(\omega ,\Psi )={\mathcal {N}}_i(\omega )=\bigoplus _{x \in Z_i(\omega )} {\widehat{\Lambda }}_{\Psi } \langle x \rangle , \end{aligned}$$

where the boundary operator $\partial$ is defined as expected: fix preferred lifts ${\hat{x}}$ of each $x \in Z(\omega )$ and define the *y*-component of $\partial x$ by the (signed) count of ${\hat{f}}_\omega$-Morse flow lines on the cover ${\widehat{M}}$ from ${\hat{x}}$ to $g \circ {\hat{y}}$ for all $g \in G$.^28^ Pajitnov proves that there always exists a $\Phi _\omega$-regular family $\Psi$, so that $({\mathcal {N}}_\bullet (\omega ),\partial )$ is a well-defined ${\widehat{\Lambda }}_\Psi$-module chain complex and shows:

#### Theorem 3.9

(Pajitnov 2019, [17]) For any Morse–Smale pair $(\omega ,g)$, there exists a $\Phi _\omega$-regular family $\Psi$, such that ${\mathcal {N}}_\bullet (\omega ,\Psi )={\mathcal {N}}_\bullet (\omega )$ is a well-defined chain complex. Moreover, for any such $\Psi$, it holds

$$\begin{aligned} {\mathcal {N}}_\bullet (\omega ) \simeq C_\bullet \left( {\widehat{M}}\right) \otimes _{{\mathbb {Z}}[G]} {\widehat{\Lambda }}_{\Psi } \end{aligned}$$

as $${\widehat{\Lambda }}_{\Psi }$$ modules.

Using Theorem 3.6, we recover Theorem 3.9 in the abelian case.

#### Corollary 3.10

Let $(\omega ,g)$ be a Morse–Smale pair, $p :{\widehat{M}} \rightarrow M$ be an abelian regular cover with $p^*[\omega ]=0$.

Then, there exist a $\Phi _\omega$-semi-regular family $\Psi$, a section $\theta :{\mathcal {A}} \rightarrow \Omega ^1(M)$ around $[\omega ]$ with ${\widetilde{M}}_{\mathcal {A}}={\widehat{M}}$, a subpolytope ${\mathcal {B}} \subseteq {\mathcal {A}}$, and a perturbation $\vartheta :{\mathcal {A}} \rightarrow \Omega ^1(M)$, such that

$$\begin{aligned} \mathrm {CN}_\bullet (\vartheta _a,{\mathcal {A}}|_{{\mathcal {B}}}) \simeq C_\bullet \left( {\widehat{M}} \right) \otimes _{{\mathbb {Z}}[G]} {\widehat{\Lambda }}_{\Psi }, \quad \forall a \in {\mathcal {A}} \end{aligned}$$

as Novikov modules with $\vartheta _{[\omega ]}=\omega$. In particular

$$\begin{aligned} \mathrm {CN}_\bullet (\omega ,{\mathcal {A}}|_{{\mathcal {B}}}) \simeq C_\bullet \left( {\widehat{M}} \right) \otimes _{{\mathbb {Z}}[G]} {\widehat{\Lambda }}_{\Psi }. \end{aligned}$$

#### Proof

First of all, we construct the polytope ${\mathcal {A}}$ and a small section $\theta :{\mathcal {A}} \rightarrow \Omega ^1(M)$ by adapting a rational approximation idea due to Pajitnov [15]; see also [21, Section 4.2]. Consider the splitting from before

$$\begin{aligned} H_1(M;{\mathbb {Z}}) \cong {\mathbb {Z}}^r \oplus \ker [\omega ], \end{aligned}$$

where *r* is the rank of $[\omega ]$. Pick *r*-many generators $\gamma _1,\dots ,\gamma _r$ of the first summand above. By de Rham’s Theorem, there are *r*-many pairwise distinct integral classes $a_1,\dots ,a_r$ dual to $\gamma _1,\dots ,\gamma _r$, such that

$$\begin{aligned} \ker (a_l) \supset \ker [\omega ], \quad \forall l=1,\dots ,r. \end{aligned}$$

In particular, we can write

$$\begin{aligned} \Phi _\omega = \sum _{l=1}^r u_l \cdot \Phi _{a_l}, \end{aligned}$$

for a unique vector ${\underline{u}}=(u_1,\dots ,u_r) \in {\mathbb {R}}^r$, and hence, $[\omega ]=\sum _{l=1}^r u_l \cdot a_l$. Thus, for our fixed Morse representative $\omega \in [\omega ]$, there exist $\alpha _l \in a_l$ with

27 $$\begin{aligned} \omega =\sum _{l=1}^r u_l \cdot \alpha _l \quad \text {and}\quad \ker [\omega ]=\bigcap _{l=1}^r \ker (a_l). \end{aligned}$$

For fixed $\varepsilon >0$, we construct *r*-many *rational* closed one-forms $\beta _l$ that are $\varepsilon$-close to $\omega$ (in the operator norm induced by *g*). Pick a sufficiently small vector ${\underline{v}}^1={\underline{v}}^1(\varepsilon ) \in {\mathbb {R}}^r$, such that for

$$\begin{aligned} \beta _1=\omega +\sum _{l=1}^r v^1_l \cdot \alpha _l, \end{aligned}$$

we have

$$\begin{aligned} \Vert \omega - \beta _1 \Vert \le \sum _{l=1}^r \vert v^1_l \vert \cdot \Vert \alpha _l \Vert < \varepsilon \quad \text {and} \quad b_1:=[\beta _1] \in H^1(M;{\mathbb {Q}}). \end{aligned}$$

This is possible, since ${\mathbb {Q}}$ is dense in ${\mathbb {R}}$. Define

$$\begin{aligned} \beta _2=\omega +\sum _{l=1}^r v_l^2 \cdot \alpha _l, \quad {\underline{v}}^2 \in {\mathbb {R}}^r, \end{aligned}$$

satisfying the same properties with

$$\begin{aligned} {\underline{v}}^2-{\underline{v}}^1=(\underbrace{v^2_1-v^1_1}_{\ne 0},0,\dots ,0). \end{aligned}$$

In particular

$$\begin{aligned} \beta _2-\beta _1=(v_1^2-v_1^1) \cdot \alpha _1, \end{aligned}$$

which implies $b_1 \ne b_2$. Proceeding inductively (e.g. ${\underline{v}}^3$ agreeing with ${\underline{v}}^2$ except for the second entry, etc.), we end up with *r*-many rational one-forms $\beta _1,\dots ,\beta _r$ satisfying

- $$\Vert \omega - \beta _j \Vert \le \sum _{l=1}^{r} \vert v^j_l \vert \cdot \Vert \alpha _l \Vert < \varepsilon $$ for all $$j=1,\dots ,r$$.
- $$b_l \ne b_j$$ for all $$l \ne j$$.
- $\ker (b_l) \supset \ker [\omega ]$ for all $l=1,\dots ,r$, by (27).

Since all $b_l$ are rational, there exists a positive integer $q \in {\mathbb {N}}$, so that every cohomology class $q \cdot b_l$ is integral. From the bullet points above, we thus conclude that

$$\begin{aligned} \Psi :=\{ \Phi _{q \cdot b_l}\} \end{aligned}$$

defines a $\Phi _\omega$-semi-regular family. Next, set ${\mathcal {A}}^-=\langle [\omega ],b_1,\dots ,b_r \rangle$ and

$$\begin{aligned} \varepsilon =\frac{C_\omega }{D \cdot 1000}, \end{aligned}$$

cf. Corollary 2.17. It is clear that there exists a section $\theta ^- :{\mathcal {A}}^- \rightarrow \Omega ^1(M)$ sending $[\omega ]$ to $\omega$ and $b_l$ to $\beta _l$. In particular

$$\begin{aligned} \Vert \theta ^-_a- \omega \Vert < \varepsilon , \quad \forall a \in {\mathcal {A}}^-. \end{aligned}$$

Analogously to the construction of the $\beta _l$’s, one can define rational one-forms close to $\omega$ that do not vanish on $\ker [\omega ]$. Including some of those cohomology classes allows us to extend ${\mathcal {A}}^-$ to ${\mathcal {A}}$, so that ${\widetilde{M}}_{{\mathcal {A}}}={\widehat{M}}$ with a section $\theta :{\mathcal {A}} \rightarrow \Omega ^1(M)$ that extends $\theta ^-$ and is still $\varepsilon$-close to $\omega$. By choice of $\varepsilon$, we can invoke Corollary 2.17 and define a perturbed section

$$\begin{aligned} \vartheta ^\omega :{\mathcal {A}} \rightarrow \Omega ^1(M) \end{aligned}$$

with $(\omega ,g)$ as the underlying reference pair. But now, we are in a position to use Theorem 3.6 and Remark 3.7 with ${\mathcal {B}}:=\langle b_1,\dots ,b_r \rangle$ to obtain

$$\begin{aligned} \mathrm {CN}_\bullet (\vartheta ^\omega _a,{\mathcal {A}}|_{{\mathcal {B}}}) \simeq C_\bullet ({\widehat{M}}) \otimes _{{\mathbb {Z}}[G]} \mathrm {Nov}({\mathcal {A}}|_{{\mathcal {B}}}) = C_\bullet ({\widehat{M}}) \otimes _{{\mathbb {Z}}[G]} \bigcap _{l=1}^r\mathrm {Nov}({\mathcal {A}}|_{b_l}). \end{aligned}$$

The last equality follows from Lemma 2.7. Note that $\mathrm {Nov}({\mathcal {A}}|_{b_l})={\widehat{{\mathbb {Z}}}}[G]^{b_l} = {\widehat{{\mathbb {Z}}}}[G]^{q \cdot b_l}$, and therefore, the Novikov ring on the RHS above does coincide with ${\widehat{\Lambda }}_{\Psi }$. By definition of the perturbation $\vartheta ^\omega$, we get $\vartheta ^\omega _{[\omega ]}=\omega$, which finally concludes the proof. $\square$

## Footnotes

1 See Corollary 3.4 for a precise statement.

2 See Theorem 2.24 for the precise statement. Also note that the actual theorem contains a stronger chain-level statement.

3 For the sake of simplicity, we omit the precise conditions.

4 This is not a circular argument, since the Novikov Morse Homology Theorem is not used in Sect. 2.

5 This is a slightly imprecise formulation; see Theorem 3.6 for the precise statement.

6 See Theorem 3.9 for a statement below.

7 Cohomologous one-forms induce the same period homomorphism by Stokes’ Theorem.

8 This is a well-known result in Riemannian geometry; see [24].

9 This is still the ball of radius $\delta$ with respect to the distance metric induced by *g*.

10 The choice of $D>0$ is independent of the assumption (7).

11 Note that here the metric is *not* scaled.

12 Explicitly, this section is of the form

$$\begin{aligned} \varepsilon \cdot a \mapsto \varepsilon \cdot \theta _a+dH - \sum _i d(f_i \cdot h_i),\end{aligned}$$

where $f_i$ depends smoothly on $\theta _a$ and satisfies $df_i=\varepsilon \cdot \theta _a, \, f_i(x_i)=0$ around critical points $x_i$ of *H*, see Lemma 2.12, (5) and (6) applied to $\varepsilon \cdot \theta _a+ dH$ and $\rho =dH$.

13 The latter is possible, since $Z(\varepsilon \cdot \vartheta _a)=Z(\varepsilon \cdot dH)=\mathrm {Crit}(H)$ is finite.

14 This is also implicitly using that ${\tilde{f}}_{\vartheta ^s_b}={\tilde{f}}_{\vartheta ^0_b}+h_s\circ \pi$ for a smooth family $h_s \in C^{\infty }(M)$, since $\vartheta ^s_b$ are cohomologous for all *s*. Hence

$$\begin{aligned} -\int _{\gamma _n} \vartheta ^s_b=-{\tilde{f}}_{\vartheta ^1_b}({\tilde{y}}_n)+{\tilde{f}}_{\vartheta ^0_b}({\tilde{x}}_n)+\underbrace{\int _{[0,1]} \frac{\partial h_s}{\partial s}(\gamma _n(s)) \, \mathrm{d}s}_{\le C}, \end{aligned}$$

for some uniform constant *C*.

15 Uniform in *b* and $n \in {\mathbb {N}}$, that is.

16 Strictly speaking, we could also use perturbations coming from Corollary 2.17 at the expanse of working with small sections. For the sake of exposition, we refrain from stating this explicitly.

17 The addition and multiplication of $\mathrm {Nov}(G)$ are the obvious ones: $n_g \, t^g+m_g \,t^g=(n_g+m_g) \, t^g$ and $(n_g \,t^g) \cdot (n_h \, t^h):=n_g \cdot n_h \, t^{g+h}$.

18 Note that $A=B$ in $\Gamma _{{\mathcal {A}}}$ if and only if $\Phi _{a_l}(A)=\Phi _{a_l}(B)$ for all $l=0,\dots ,k$.

19 This stems from the construction of a category equivalence between the fundamental groupoid and the fundamental group of a sufficiently nice topological space. This procedure allows to switch back and forth between local coefficients and representations, see [10, Page 17], [1, Chapter 2] and [3] for more details.

20 Note that $$\Gamma (\gamma * \eta )=\Gamma (\gamma ) \circ \Gamma (\eta )$$.

21 Note that if 0 is already contained in ${\mathcal {A}}$, then ${\mathcal {A}}^0={\mathcal {A}}$.

22 We are implicitly identifying $\Lambda _{{\mathcal {A}} |_{{\mathcal {B}}}}=\mathrm {Nov}({\mathcal {A}} |_{{\mathcal {B}}})$, see Proposition 2.27.

23 Sanity check: $\lambda _{m,n}=\sum _{{\mathcal {A}} \in \Gamma _{{\mathcal {A}}}} \#_{\mathrm {alg}} \, \underline{{\mathcal {M}}}({\tilde{x}}_m,A {\tilde{y}}_n; {\tilde{h}}) \, A$, but $\bigcup _{A \in \Gamma _{{\mathcal {A}}}}\underline{{\mathcal {M}}}({\tilde{x}}_m,A {\tilde{y}}_n;{\tilde{h}}) \cong \underline{{\mathcal {M}}}(x_m,y_n;h)$ and the latter is finite.

24 The authors mention in the proof of [1, Lemma 6.30] that the isomorphism in [1, Theorem 4.1] is an isomorphism in the category of the underlying local coefficient system, thus a Novikov-module isomorphism in our case.

25 See the proof of [1, Theorem 3.9] for more details.

26 One can also deduce from category theory by observing that the functor $F :\mathsf {mod}_{{\mathbb {Z}}[{\mathcal {A}}]} \rightarrow \mathsf {mod}_{\mathrm {Nov}({\mathcal {A}} |_{{\mathcal {B}}})}$,

$$\begin{aligned} F(O):=O \otimes _{{\mathbb {Z}}[\Gamma _{{\mathcal {A}}}]} \mathrm {Nov}({\mathcal {A}} |_{{\mathcal {B}}}),\, F(i)=i \otimes \mathrm {id}, \quad O,P \in \mathsf {obj}\left( \mathsf {mod}_{{\mathbb {Z}}[\Gamma _{{\mathcal {A}}}]}\right) , \, i \in \hom (O,P) \end{aligned}$$

is additive.

27 The rank of a cohomology class $a \in H^1_{\mathrm {dR}}(M)$ is defined as $\mathrm {rank}_{{\mathbb {Z}}}\left( \mathrm {im} \, \Phi _a\right)$.

28 The definition in [17] is slightly more general, as they define the count with respect to any transverse $\omega$-gradient.
